# Vibronic Exciton–Phonon States in Stack-Engineered van der Waals Heterojunction Photodiodes

**DOI:** 10.1021/acs.nanolett.2c00944

**Published:** 2022-07-05

**Authors:** Fatemeh Barati, Trevor B. Arp, Shanshan Su, Roger K. Lake, Vivek Aji, Rienk van Grondelle, Mark S. Rudner, Justin C. W. Song, Nathaniel M. Gabor

**Affiliations:** ^†^Laboratory of Quantum Materials Optoelectronics, ^‡^Department of Physics and Astronomy, and ^§^Laboratory for Terahertz and Terascale Electronics (LATTE), Department of Electrical and Computer Engineering, University of California—Riverside, Riverside, California 92521, United States; ∥ Department of Physics and Astronomy, Faculty of Sciences, Vrije Universiteit Amsterdam, De Boelelaan 1081, 1081 HV Amsterdam, The Netherlands; ⊥ Canadian Institute for Advanced Research, MaRS Centre West Tower, 661 University Avenue, Toronto, Ontario ON M5G 1M1, Canada; # Department of Physics, University of Washington, Seattle, Washington 98195, United States; ∇ Niels Bohr Institute, University of Copenhagen, 2200 Copenhagen, Denmark; • Division of Physics and Applied Physics, School of Physical and Mathematical Sciences, Nanyang Technological University, Singapore 637371, Singapore

**Keywords:** vibronic, photocurrent, interlayer excitons, stack engineering, van der Waals heterostructures

## Abstract

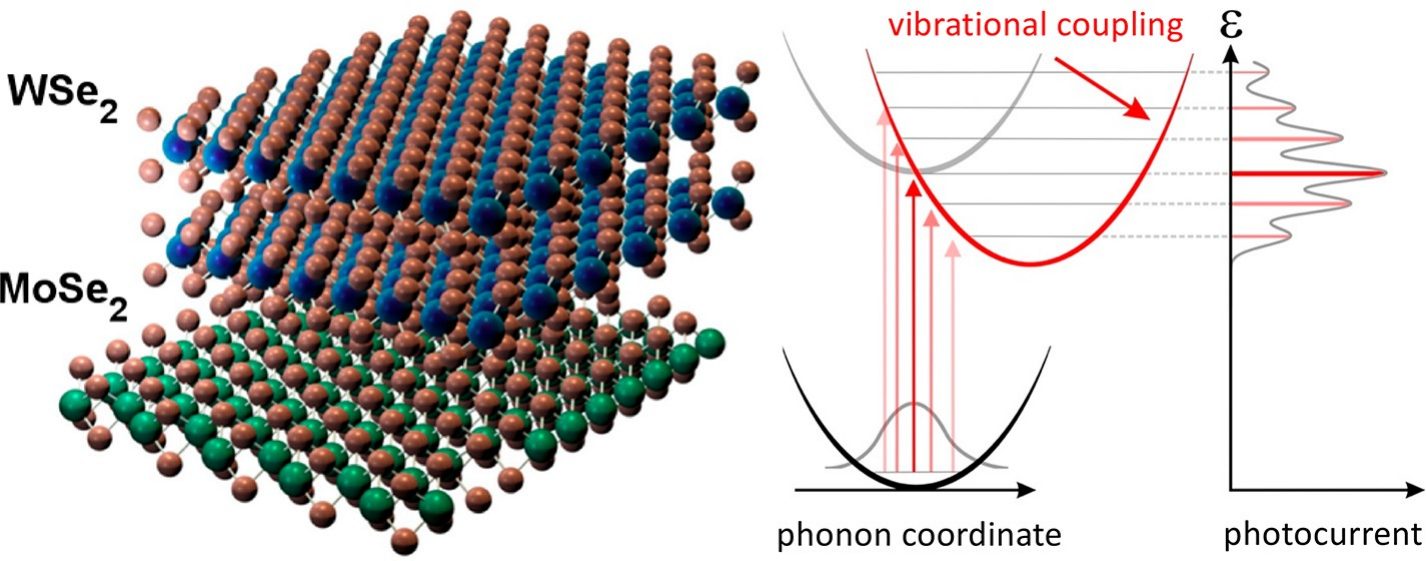

Stack engineering, an atomic-scale metamaterial strategy, enables the design of optical and electronic properties in van der Waals heterostructure devices. Here we reveal the optoelectronic effects of stacking-induced strong coupling between atomic motion and interlayer excitons in WSe_2_/MoSe_2_ heterojunction photodiodes. To do so, we introduce the photocurrent spectroscopy of a stack-engineered photodiode as a sensitive technique for probing interlayer excitons, enabling access to vibronic states typically found only in molecule-like systems. The vibronic states in our stack are manifest as a palisade of pronounced periodic sidebands in the photocurrent spectrum in frequency windows close to the interlayer exciton resonances and can be shifted “on demand” through the application of a perpendicular electric field via a source-drain bias voltage. The observation of multiple well-resolved sidebands as well as their ability to be shifted by applied voltages vividly demonstrates the emergence of interlayer exciton vibronic structure in a stack-engineered optoelectronic device.

The vibrational motion of atoms in solids is ordinarily expected to dissipate energy from electronic excitations. Strong coupling of vibrational motion to electrons, however, can significantly transform the nature of accessible excited states, dramatically enriching light–matter interactions. In soft matter, such as photosynthetic light-harvesting complexes, the interplay between atomic motion and exciton dynamics enhances electronic energy transfer^1−4^ in spite of the fluctuating physical environment. In crystals, the presence of strong interactions between electronic excitations and phonons,^5,6^ which are the elementary excitations of the atomic lattice, enables the trapping of excitations,^7−9^ allows mechanical control of electron transport,^10,11^ and drives the formation of exotic exciton–phonon quasiparticles and excitonic complexes.^12,13^

A particularly striking manifestation of strong exciton–phonon coupling is the periodic vibronic structure that appears in molecular absorption spectra.^14^ The well-separated and multiple peaks in these systems correspond to distinct vibronic states; resonant excitation therefore enables us to directly address each individual state. Easy access to both optical and electronic control in transition-metal dichalcogenides (TMDs) offers a unique opportunity to electrically control vibronic states in a semiconductor device. Nevertheless, the voltage-tunable vibronic structure of periodic and well-separated peaks has not been realized in semiconductor TMD heterostructure devices.

Here we report the emergence of multiple periodic and well-separated photocurrent peaks when individual van der Waals (vdW) layers are stacked to form atomically thin heterostructure photodiodes. In particular, we find that the interlayer photocurrent exhibits a rich structure with numerous photocurrent sidebands as a function of incident photon energy *E*_PH_. Strikingly, photocurrent sidebands are manifested only for values of *E*_PH_ close to the interlayer exciton energy; they are absent for the intralayer excitons. These resonances occur periodically with an energy spacing of approximately 30 meV and are observed for two distinct interlayer excitons within the same heterostructure. This energy spacing corresponds to the frequency of a group of prominent phonon modes observed in the Raman spectrum of the heterostructure. Importantly, we demonstrate that the manifold of photocurrent peaks can be controlled by the source-drain bias voltage.

As we discuss below, this multipeaked structure of the exciton resonance can be attributed to the appearance of a manifold of well-formed vibronic coupled exciton–phonon states (mirroring those typically found in molecular systems). We note that this vibronic structure of interlayer excitons in vdW heterostructures has so far been obscured from purely optical probes such as photoluminescence (PL);^15^ see also the discussion below. In contrast, the multicomponent photocurrent spectroscopy that we employed provides a means to directly address and control the rich vibronic structure of interlayer excitons that results from strong electron–phonon coupling in stacked vdW materials.

Experimentally, we studied vdW heterostructures composed of bilayer tungsten diselenide (WSe_2_) stacked on top of monolayer molybdenum diselenide (MoSe_2_), as shown in Figure 1. *2L*-WSe_2_/MoSe_2_ serves as an important benchmark for photocurrent spectroscopy because it exhibits two distinct interlayer exciton resonances, one which has been observed in many PL studies of WSe_2_/MoSe_2_ (*K* → *K*) and another that has not been previously observed (Γ → *K*), as discussed in detail below. Owing to this newly observed interlayer exciton and the type II band alignment (see detailed band structure calculations in ref (25)), *2L*-WSe_2_/MoSe_2_ also fulfills the technological need for semiconductor heterostructures with near-infrared band gaps, similar to silicon. Indeed, this work establishes it as an excellent material system with a clearly evident photocurrent near 1 eV.

**Figure 1:**
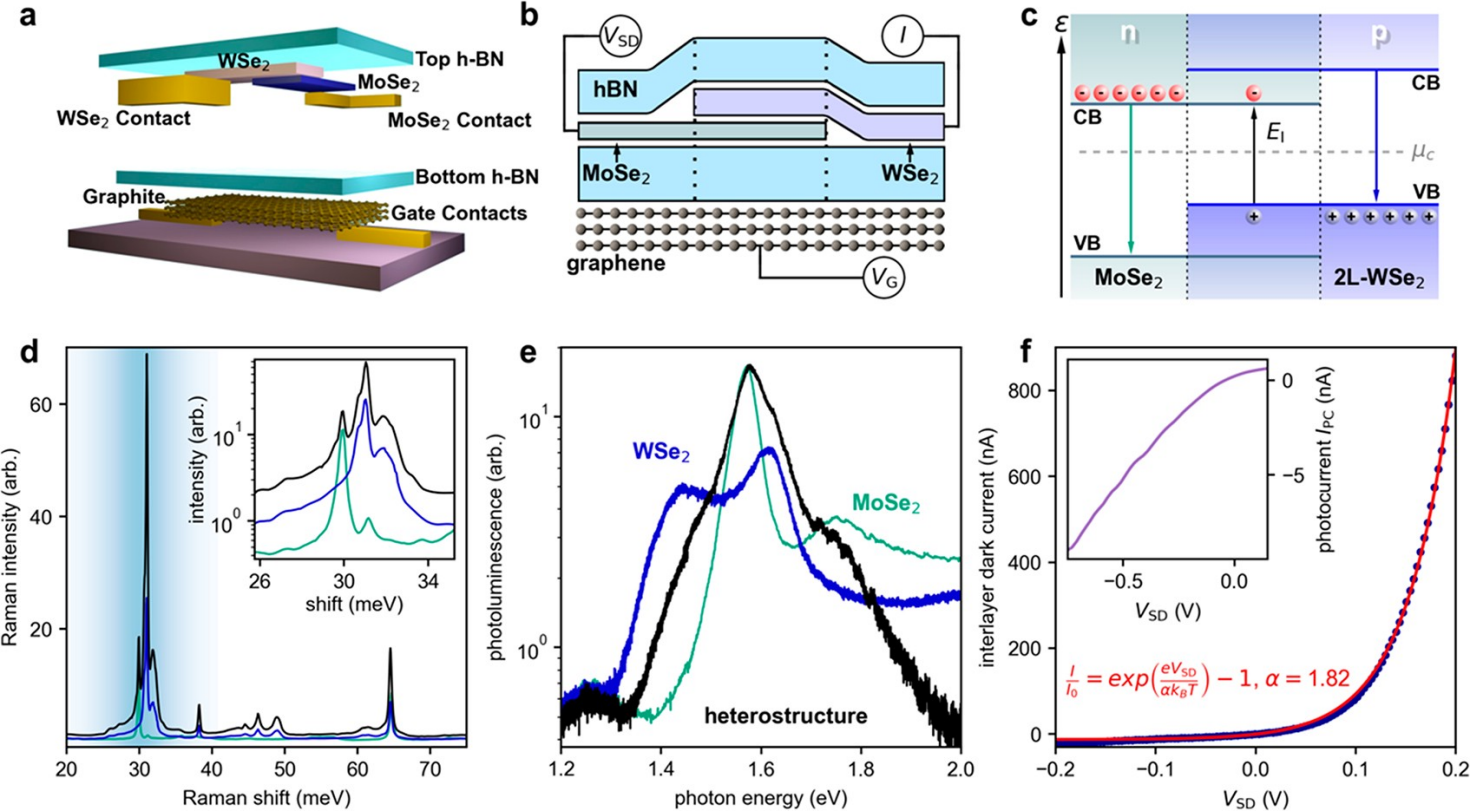
Stack engineering and characterization of encapsulated WSe_2_-MoSe_2_ heterojunction photodiode devices. (a) Schematic of the multistage inverted fabrication process. (b) Schematic of the hexagonal boron nitride (hBN)-encapsulated heterostructure with a multilayer graphene back gate and source-drain electrodes. (c) Electronic energy band diagram at *V*_SD_ = 0 V, showing the conduction bands (CB), valence bands (VB), and chemical potential *μ*_c_ in equilibrium (horizontal dashed gray line). (d) Raman spectroscopy (λ = 532 nm) of MoSe_2_ (green), bilayer WSe_2_ (blue), and the heterostructure (black). (Inset) log-scale Raman intensity versus Raman shift near 30 meV. (e) Photoluminescence (λ = 532 nm) from MoSe_2_ (green), bilayer WSe_2_ (blue), and the heterostructure (black). (f) Interlayer dark current vs *V*_SD_ (blue points); the device displays ordinary vdW heterostructure p–n junction behavior^24−27^ with a fit (solid red line) to the diode using ideality factor α = 1.82, which relates the applied voltage *V*_SD_ to the potential energy difference established across the WSe_2_–MoSe_2_ interface.^17,29^ (Inset) Interlayer photocurrent vs *V*_SD_; *E*_PH_ = 0.99 eV. (See the Supporting Information Section S4(16) for details.)

Using an inverted fabrication process (Figure 1a, Supporting Information Section S1(16)), we first patterned multilayer graphene gate electrodes and conventional metal source and drain contacts. The WSe_2_–MoSe_2_ heterostructures, assembled and characterized independently, were laminated onto the prefabricated device patterns. Combining the vdW heterostructure in this way enabled complete protective encapsulation of the WSe_2_-MoSe_2_ interface region between hexagonal boron nitride layers (schematic in Figure 1b). We fabricated and studied three devices, each with a random orientation between constituent layers. The results described below were consistent across all devices.

Devices were characterized using Raman, photoluminescence (PL), and photocurrent (PC) spectroscopy. As shown in Figure 1d, the Raman spectrum of the heterostructure (black line) exhibits peaks that are also evident in the individual MoSe_2_ (green line) and WSe_2_ (blue line) layers. When comparing Raman peaks between the heterostructure and its constituent layers, we observed a negligible shift of the peak positions as a function of energy. Importantly, we observed pronounced Raman peaks at the WSe_2_-MoSe_2_ interface (black line), at energies near 30 meV (29.9, 31.0, and 31.9 meV, Figure 1d inset). In our previous work,^17^ Raman peaks near 30 meV were used to identify the layer thickness, confirming that the heterostructure was composed of bilayer WSe_2_ and monolayer MoSe_2_. In MoSe_2_, the A_1g_ peak at 241 cm^–1^ (29.8 meV), E_2g_^1^ peak at 288 cm^–1^, and lack of B_2g_^1^ peak between 350 and 360 cm^–1^ are characteristic of monolayer thickness. The Raman spectrum of WSe_2_ exhibits an A_1g_ mode at 250 cm^–1^ (30.9 meV), an E_2g_ mode at 260 cm^–1^ (32.2 meV), and a B_2g_^1^ mode at 309 cm^–1^, indicating bilayer thickness.

Figure 1e compares the PL vs photon energy *E*_PH_ from MoSe_2_ (green line), WSe_2_ (blue line), and the heterostructure (black line). The stacked vdW heterostructure exhibits PL spectral features similar to those of the individual layers.^18−23^ In addition to the observed excitonic resonances characteristic of the individual layers, low-energy bound interlayer e–h pairs (excitons) are known to form between the valence band of bilayer WSe_2_ and the conduction band of MoSe_2_ (Figure 1c). Direct access to interlayer excitons has proven to be challenging. For example, although signatures of the lowest-lying interlayer exciton near *E*_I_ ≈ 1.0 eV (arising from carriers in the momentum mismatched Γ- and *K*-valleys) can be indirectly inferred,^17^ direct PL signatures are washed out as a result of very small oscillator strengths.

To overcome the small oscillator strength that prevents strong and direct PL signatures for these interlayer excitons, here we instead employed measurements of the interlayer photocurrent *I*_PC_ with the laser focused on the WSe_2_-MoSe_2_ heterostructure. In the photocurrent setup, interlayer excitons that are generated by infrared laser illumination of the vdW heterostructure can be subsequently dissociated. Once dissociated, separated electrons and holes transit the device, resulting in a photocurrent that increases in reverse bias (Figure 1f inset; the main panel shows dark current characterization of the device, displaying typical diode behavior).^23−27^

Figure 2 examines the detailed dependence of the interlayer photocurrent on *E*_PH_ and *V*_G_. Using sensitive current amplification and sweeping *E*_PH_, we find that *I*_PC_, which is the difference in current measured with the light on and light off, is particularly pronounced in two frequency windows occurring near *E*_PH_ ≈ 1.3 and 0.9 eV. Photoexcitation energies in the vicinity of these two hot spots correspond to interlayer excitons hosted in the WSe_2_-MoSe_2_ heterostructure, namely, the *K* → *K* (∼1.3 eV) and Γ → *K* (∼0.9 eV) interlayer excitons.^17,18,28^

**Figure 2:**
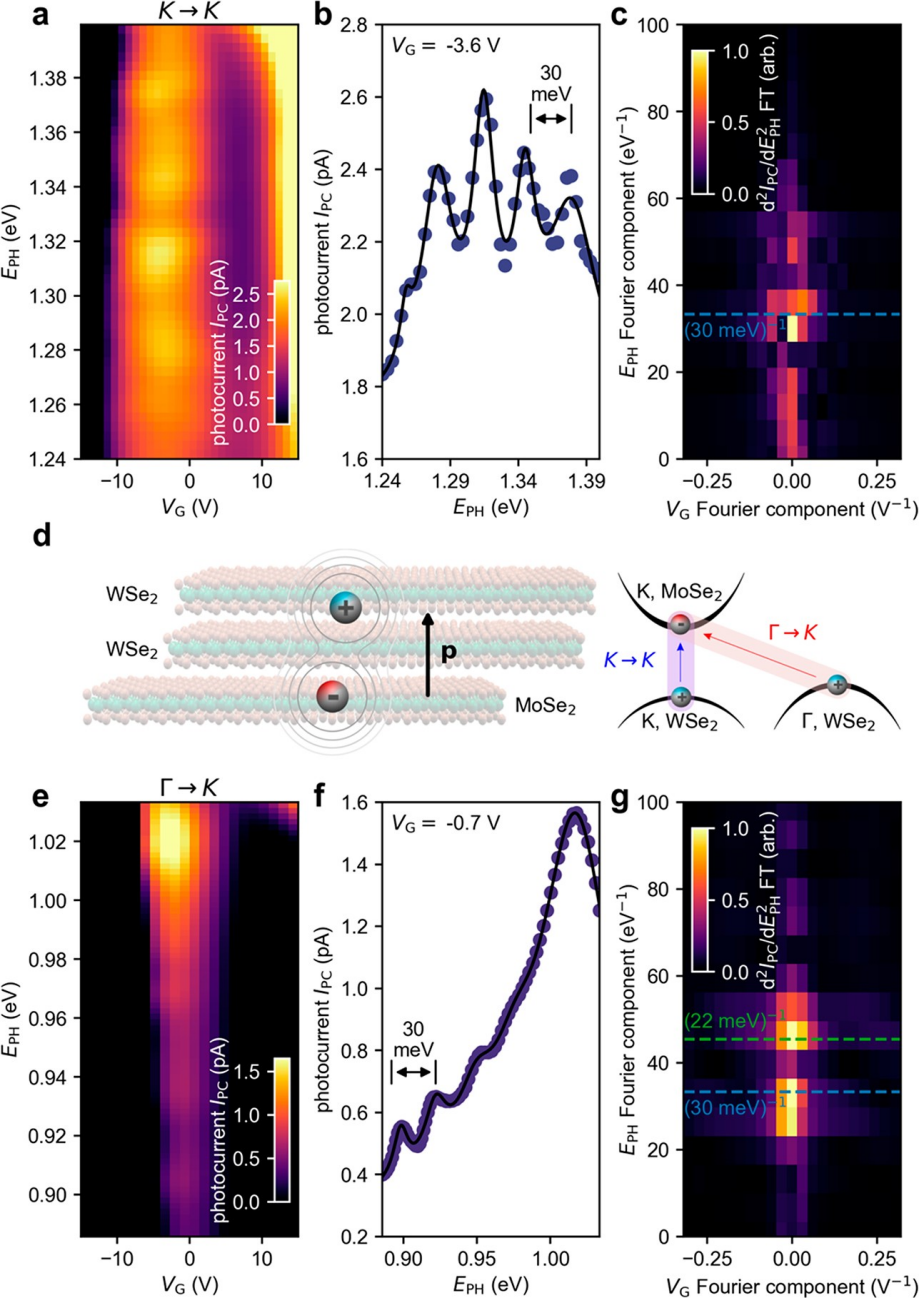
Multiple and periodic sidebands in the photocurrent spectra of van der Waals p–n heterojunction devices. (a) Interlayer photocurrent *I*_PC_ vs *E*_PH_ and *V*_G_ near the direct (*K* → *K*) interlayer exciton transition for device 1, *T* = 20 K. (b) Line cut of the photocurrent spectrum at *V*_G_ = −3.5 V. For full data, see Supporting Information Section S4.2.^16^ (c) 2D Fourier transform of the photocurrent vs *E*_PH_ second derivative. (d, left) Schematic of the interlayer exciton in the heterostructure; (right) schematic of the band structure with the momentum direct (*K* → *K*) and indirect (Γ → *K*) interlayer excitons labeled. (e) *I*_PC_ vs *E*_PH_ and *V*_G_ of the heterostructure near the Γ → *K* exciton at *T* = 20 K. (f) Line cut of the photocurrent spectrum. (g) 2D Fourier transform of the photocurrent vs *E*_PH_ second derivative.

Strikingly, when *E*_PH_ was tuned between 1.24 and 1.40 eV (Figure 2a), we observed a periodic sequence of photocurrent peaks that occur in a narrow range of gate voltages (near *V*_G_ = −3.5 V). Although the strongest peak occurs at *E*_PH_ = 1.32 eV, it is only slightly stronger than several equally spaced maxima at higher and lower *E*_PH_ (Figure 2b). We observed an average peak separation of 30 meV. To clarify this periodic modulation, we calculated the Fourier transform of the second derivative of the photocurrent data, where we find a clear periodic component at 1/Δε = (30 meV)^−1^ (marked by the blue dashed line in Figure 2c). Interestingly, near the interlayer excitation from the WSe_2_*K*-valley to the MoSe_2_*K*-valley (Figure 2d), the discrete energy difference Δε = 30 meV between photocurrent peaks closely corresponds to the frequency at which a strong Raman signal is observed in the heterojunction, ℏΩ ≈ 30 meV. Here, ℏ is Planck’s constant and Ω is the phonon frequency.

In the same fashion as above, optical excitation of the lowest-lying (Γ → *K*) interlayer exciton also results in a series of approximately equally spaced discrete sidebands with energy spacing ℏΩ ≈ 30 meV. This behavior is highlighted in Figure 2e, which shows *I*_PC_ vs *E*_PH_ and *V*_G_ at infrared photon energies. In the range *E*_PH_ = 0.88–1.03 eV, we observed a set of evenly spaced photocurrent maxima, which increase in amplitude as *E*_PH_ increases. The lowest-energy peak occurs at *E*_PH_ = 0.90 eV, and line traces of *I*_PC_ vs *E*_PH_ (Figure 2f) show regularly spaced peaks that are superimposed on a photocurrent background that increases with *E*_PH_. Taking the Fourier transform of the second derivative of the interlayer photocurrent data (Figure 2g) reveals two periodic components: a dominant component at Δε = 30 meV and a weaker component at 22 meV. In sharp contrast to this periodic structure in Figure 2, photocurrent corresponding to the excitation of intralayer excitons does not produce such sidebands (Supporting Information Section S4.1^16^).

The appearance of a periodic array of photocurrent sidebands measured at fixed source-drain bias *V*_SD_ can be understood through the strong coupling of phonons and interlayer excitons, with each of the sideband peaks identified with a coupled (interlayer) exciton–phonon state of frequency *ω*_vib,*n*_ (*V*_SD_), where *n* is an index that labels the vibronic states/peaks. In these vibronic states, electronic excitations are intertwined with lattice displacements and can be understood as Franck–Condon-type progressions. (See the detailed theoretical description in Supporting Information Section S5.^16^) We note, parenthetically, that the 30 meV periodicity in photocurrent spectroscopy (Figure 2) closely matches a window of narrow phonon branches expected for the interlayer heterostructure. (See Supporting Information Section S3(16) for discussion of phononic origins as well as phonon dispersion calculations in the heterostructure.) Remarkably, each of these vibronic states, *ω*_vib,*n*_, is addressable by tuning *E*_PH_. This contrasts with phonon-broadened peaks wherein the action of exciton–phonon interaction broadens the exciton transition without yielding individual well-defined exciton–phonon states.^6^

Although the *E*_PH_ spectroscopy in Figure 2 revealed the vibronic structure (at fixed *V*_SD_), we can leverage the out-of-plane electric field of the interlayer p–n junction to electrically control the registry of vibronic states. To see this, we conducted detailed *V*_SD_-dependent measurements of the interlayer exciton photoresponse. Figure 3a shows photoconductance (d*I*_PC_/d*V*_SD_) maps as a function of *V*_SD_ and *V*_G_ for a reverse-biased p–n heterojunction (device 2) with *E*_PH_ = 0.99 eV. In these d*I*_PC_/d*V*_SD_ maps, multiple vertical stripes stand out, corresponding to prominent periodic oscillations (as a function of *V*_SD_) in the photoconductance (Figure 3b). Indeed, this periodic oscillation in photoconductance is confirmed via Fourier transform of the d*I*_PC_/d*V*_SD_ maps and displays a well-defined *V*_SD_ periodicity. These photoconductance oscillations, observed at room temperature, can be understood to arise from *V*_SD_ shifting the entire registry of vibronic peaks so that each peak shifts in and out of resonance with the fixed *E*_PH_.

**Figure 3:**
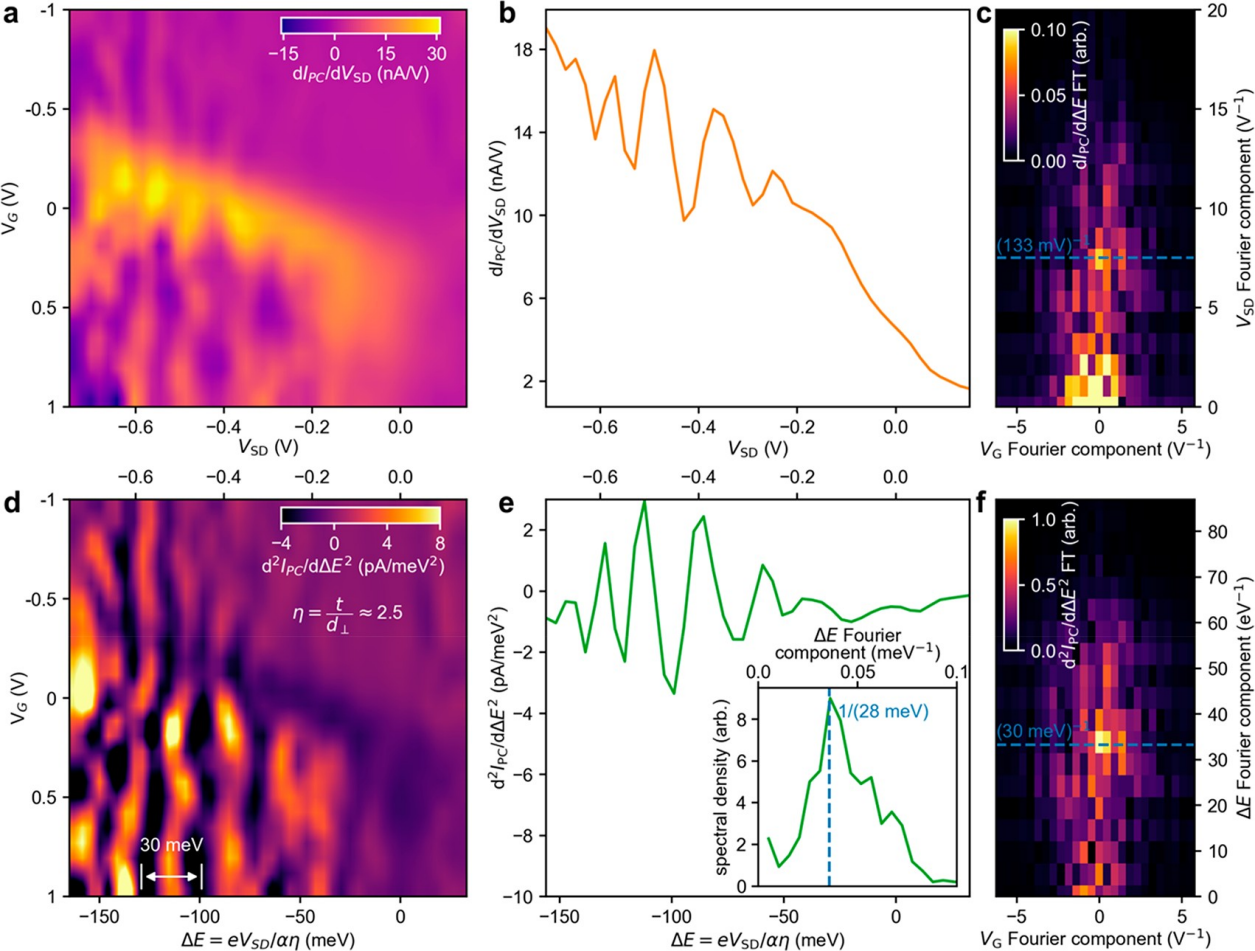
Electrical control of the vibronic photoresponse in WSe_2_-MoSe_2_ heterojunction devices. (a) Interlayer photoconductance d*I*_PC_/d*V*_SD_ vs *V*_SD_ and *V*_G_ for device 2 (*E*_PH_ = 0.99 eV) at room temperature. Data were extracted from a large set of spatial photocurrent maps to isolate the WSe_2_-MoSe_2_ heterostructure photoresponse (Supporting Information Section S4^16^). (b) Photoconductance vs *V*_SD_ line trace extracted from the photoconductance map (averaged over the peak response region between *V*_G_ = −0.3 and 0.4 V). (c) 2D Fourier transform of the data in a. (d) Color map of d^2^*I*_PC_/d*ΔE*^2^ vs *ΔE* and *V*_G_. Δ*E* = *eV*_SD_/*αη* is determined by combining the phenomenological factors extracted from the data in Figure 1f with the dimensionless parameter η = *t*/*d*_⊥_. *V*_SD_ values are shown on the top axis. (e) d^2^*I*_PC_/d*ΔE*^*2*^ versus *ΔE*. (Inset) Fourier transform spectral density of the Fourier transformed and averaged data from a. (f) 2D Fourier transform of the second-derivative data in a. Additional details are in Supporting Information Sections S2.2 and S4.3.^16^

Electrical control of the vibronic peaks is consistent with the fact that interlayer excitons exhibit an electric dipole, **p**, that points out of plane. The exciton–phonon vibronic states can therefore naturally be sensitive to an out-of-plane electric field, **E**_*i*_. Indeed, the electrical control of *ω*_vib,*n*_ (*V*_SD_) can be understood through a Stark shift Δ*E* = **p**·**E**_*i*_ = *ed*_⊥_*V*_SD_/*tα* = *eV*_SD_/*αη* that uniformly shifts the registry of vibronic states. Here, η = *t*/*d*_⊥_ is a dimensionless quantity that compares the interlayer distance *t* to the effective dipole length *d*_⊥_ (assumed to be approximately the same for all states). The ideality factor α (obtained in fits of Figure 1f) appears in this expression to phenomenologically capture the reduction of *V*_SD_ due to leakages such as nonideal contacts.^29^ In the vdW p–n junction photodiode, the Stark shift and thus the registry of vibronic states are directly controlled using *V*_SD_.

Utilizing the *V*_SD_-dependent photoresponse to probe interlayer vibronic states, as demonstrated here, differs strongly from previous experiments on vdW heterostructures. Recently, interlayer exciton dipole strengths were estimated by studying an electrically controlled Stark shift through purely optical signals,^30,31^ and no evidence of a vibronic registry was reported. In our p–n junction photodiode, we can utilize the *V*_SD_-controlled optoelectronic response to estimate the dipole strength. As an illustration, we note that the separation between periodic vibronic peaks in Figure 2f approximately corresponds to 30 meV, yielding a modest estimate η = *t*/*d*_⊥_ ≈ 2.5 (consistent with recent measurements of interlayer exciton dipole strengths^34^). This yields energy-dependent d^2^*I*_PC_/dΔ*E*^*2*^ vs Δ*E* oscillations with a 30 meV increment (Figure 3d,f). In these (Figure 3e), each pronounced dip corresponds to a vibronic state *ω*_vib,*n*_ (*V*_SD_).

These results also differ from previous experiments on individual monolayer vdW materials. Although the absorption spectra of monolayer TMDs generally display broad absorption peaks,^31−34^ optical measurements of some vdW materials have attributed sparse and isolated sidebands to individual vibrational modes.^35−39^ In MoSe_2_^40,41^ and WSe_2_,^42,43^ optical spectroscopy measurements have resolved sideband features attributed to strong intralayer exciton–phonon coupling yet reported only at very low temperatures. Here, photoconductance oscillations are clearly observed at room temperature (Figure 3), indicating that the photocurrent is a sensitive probe of interlayer excitons, which are particularly susceptible to exciton–phonon coupling that emerges from the stacking of two vdW crystal layers. Combining the photocurrent with other advanced optical and optoelectronic techniques may allow the further elucidation of the fine details of the electron–phonon coupling in the heterostructure (e.g., distinguishing adiabatic^5^ and nonadiabatic/resonant couplings^44^) and the measurement of its strength (e.g., determining whether the vibronic coupling is strong enough to induce self-trapping of the excitons^7,9^). Such advanced techniques could include coherent optical spectroscopy such as 2D electronic spectroscopy or methods to enhance the observation and identification of relevant phonons (e.g., resonant Raman).

By stacking atomically thin semiconductors, we have demonstrated a new type of device that harnesses both vibrational and electronic energy to absorb near-infrared light, an important part of the solar light spectrum. In solid-state optoelectronics, atomic vibrations are often thought of as a loss mechanism. For example, the excess energy of photoexcited electrons above the band edge is typically lost to phonons, thus lowering the efficiency of solar cells. This paradigm is turned on its head in photosynthetic complexes, wherein atomic vibrations are instead harnessed to enhance energy transport through strong vibronic coupling. Our device, a rudimentary vibronic photodiode, exhibits vibronic effects that are often observed in photosynthesis yet have not been harnessed in solid-state devices. Stack engineering, exemplified here by stacking two vdW layers, MoSe_2_ and WSe_2_, gives rise to an entire registry of exciton–phonon vibronic states that can be individually addressed and electrically controlled in a solid-state setting. From a broader perspective, access to vibronic states in these vdW layers may enable excitonic phenomena more traditionally found in molecule-like systems, ranging from singlet fission^45^ and exciton dissociation^46^ in organic compounds to long-lived coherent dynamics and enhancements to exciton transport in photosynthetic complexes.^1−4,47−49^ We anticipate that stack engineering of strong exciton–phonon coupling will establish vdW heterojunctions as a versatile platform for controlling vibronic physics in 2D semiconductors devices.

## Supporting Information Available

Detailed description of the three experimental techniques used to identify vibronic exciton–phonon states; numerical calculations of the phonon dispersion within the heterostructure; several candidate modes and detailed microscopic descriptions of atomic vibrations; additional photocurrent spectroscopy and scanning photocurrent data analysis; and a review of the theory of Franck–Condon transitions (PDF)

## References

[ref1] Lee H.; Cheng Y.; Fleming G. R. Coherence dynamics in photosynthesis: protein protection of excitonic coherence. Science 2007, 316, 1462–1465. 10.1126/science.1142188. 17556580

[ref2] Mohseni M.; Rebentrost P.; Lloyd S.; Aspuru-Guzik A. Environment-assisted quantum walks in photosynthetic energy transfer. J. Chem. Phys. 2008, 129, 174106 10.1063/1.3002335. 19045332

[ref3] Collini E.; Wong C. Y.; Wilk K. E.; Curmi P. M. G.; Brumer P.; Scholes G. D. Coherently wired light-harvesting in photosynthetic marine algae at ambient temperature. Nature 2010, 463, 644 10.1038/nature08811. 20130647

[ref4] Ma F.; Romero E.; Jones M. R.; Novoderezhkin V. I.; Van Grondelle R. Both electronic and vibrational coherences are involved in primary electron transfer in bacterial reaction center. Nat. Commun. 2019, 10, 933 10.1038/s41467-019-08751-8. 30804346 PMC6389996

[ref5] Toyozawa Y. Optical Processes in Solids; Cambridge University Press: Cambridge, U.K., 2003.

[ref6] Perebeinos V.; Tersoff J.; Avouris P. Effect of Exciton-Phonon Coupling in the Calculated Optical Absorption of Carbon Nanotubes. Phys. Rev. Lett. 2005, 94, 027402 10.1103/PhysRevLett.94.027402. 15698227

[ref7] Rashba E. I. Excitons; North-Holland Publishing Company: Amsterdam, 1982; Chapter 13, pp 543–597.

[ref8] Grover M. K.; Silbey R. Exciton-Phonon interactions in molecular crystals. J. Chem. Phys. 1970, 52, 2099 10.1063/1.1673263.

[ref9] Toyozawa Y. Phonon structures in the spectra of solids. J. Lumin. 1970, 1, 736–746.

[ref10] Koch J.; von Oppen F. Franck-Condon blockade and giant Fano factors in transport through single molecules. Phys. Rev. Lett. 2005, 94, 206804 10.1103/PhysRevLett.94.206804. 16090269

[ref11] Leturcq R.; Stampfer C.; Inderbitzin K.; Durrer L.; Hierold C.; Mariani E.; Schultz M. G.; von Oppen F.; Ensslin K. Franck-Condon blockade in suspended carbon nanotube quantum dots. Nat. Phys. 2009, 5, 327–331. 10.1038/nphys1234.

[ref12] Toyozawa Y.; Hermanson J. Exciton-phonon bound state: a new quasiparticle. Phys. Rev. Lett. 1968, 21, 1637 10.1103/PhysRevLett.21.1637.

[ref13] Itoh T.; Nishijima M.; Ekimov A. I.; Gourdon C.; Efros AI. L.; Rosen M. Polaron and exciton-phonon complexes in CuCl Nanocrystals. Phys. Rev. Lett. 1995, 74, 1645 10.1103/PhysRevLett.74.1645. 10059081

[ref14] Harris D. C.; Bertolucci M. D. Symmetry and Spectroscopy: An Introduction to Vibrational and Electronic Spectroscopy; Dover Publications: New York, 1989.

[ref15] Rivera P.; Yu H.; Seyler K.; Wilson N. P.; Yao W.; Xu X. Interlayer valley excitons in heterobilayers of transition metal dichalcogenides. Nat. Nanotechnol. 2018, 13, 1004–1015. 10.1038/s41565-018-0193-0. 30104622

[ref16] See the Supporting Information for a discussion of the device fabrication, experimental details, a discussion of phonon modes, as well as a brief theoretical description of Franck–Condon progressions. Replication Data can be found in ref (50). The Supporting Information also contain refs (51−64).

[ref17] Barati F.; Grossnickle M.; Su S.; Lake R. K.; Aji V.; Gabor N. M. Hot carrier-enhanced interlayer electron–hole pair multiplication in 2D semiconductor heterostructure photocells. Nat. Nanotechnol. 2017, 12, 1134–1139. 10.1038/nnano.2017.203. 28991242

[ref18] Karni O.; Barré E.; Lau S. C.; Gillen R.; Ma E. Y.; Kim B.; Watanabe K.; Taniguchi T.; Maultzsch J.; Barmak K.; Page R. H.; Heinz T. F. Infrared Interlayer Exciton Emission in MoS2/WSe2 Heterostructures. Phys. Rev. Lett. 2019, 123, 247402 10.1103/PhysRevLett.123.247402. 31922842

[ref19] Unuchek D.; Ciarrocchi A.; Avsar A.; Watanabe K.; Taniguchi T.; Kis A. Room-temperature electrical control of exciton flux in a van der Waals heterostructure. Nature 2018, 560, 340–344. 10.1038/s41586-018-0357-y. 30046107

[ref20] Rivera P.; Seyler K. L.; Yu H.; Schaibley J. R.; Yan J.; Mandrus D. G.; Yao W.; Xu X. Valley-polarized exciton dynamics in a 2D semiconductor heterostructure. Science 2016, 351, 688–691. 10.1126/science.aac7820. 26912854

[ref21] Rivera P.; Schaibley J. R.; Jones A. M.; Ross J. S.; Wu S.; Aivazian G.; Klement P.; Seyler K.; Clark G.; Ghimire N. J.; Yan J.; Mandrus D. G.; Yao W.; Xu X. Observation of long-lived interlayer excitons in monolayer MoSe_2_–WSe_2_ heterostructures. Nat. Commun. 2015, 6, 6242 10.1038/ncomms7242. 25708612

[ref22] Rigosi A. F.; Hill H. M.; Li Y.; Chernikov A.; Heinz T. F. Probing interlayer interactions in transition metal dichalcogenide heterostructures by optical spectroscopy: MoS2/WS2 and MoSe2/WSe2. Nano Lett. 2015, 15, 5033–5038. 10.1021/acs.nanolett.5b01055. 26186085

[ref23] Furchi M. M.; Pospischil A. A.; Libisch F.; Burgdörfer J.; Mueller T. Photovoltaic effect in an electrically tunable van der Waals heterojunction. Nano Lett. 2014, 14, 4785–4791. 10.1021/nl501962c. 25057817 PMC4138224

[ref24] Lee C.; Lee G.; van der Zande A. M.; Chen W.; Li Y.; Han M.; Cui X.; Arefe G.; Nuckolls C.; Heinz T. F.; Guo J.; Hone J.; Kim P. Atomically thin p–n junctions with van der Waals heterointerfaces. Nat. Nanotechnol. 2014, 9, 676–681. 10.1038/nnano.2014.150. 25108809

[ref25] Ross J.; Rivera P.; Schaibley J.; Lee-Wong E.; Yu H.; Taniguchi T.; Watanabe K.; Yan J.; Mandrus D.; Cobden D.; Yao W.; Xu X. Interlayer exciton optoelectronics in a 2D heterostructure p–n junction. Nano Lett. 2017, 17, 638–643. 10.1021/acs.nanolett.6b03398. 28006106

[ref26] Buscema M.; Island J. O.; Groenendijk D. J.; Blanter S. I.; Steele G. A.; van der Zant H. S. J.; Castellanos-Gomez A. Photocurrent generation with two-dimensional van der Waals semiconductors. Chem. Soc. Rev. 2015, 44, 3691–3718. 10.1039/C5CS00106D. 25909688

[ref27] Frisenda R.; Molina-Mendoza A. J.; Mueller T.; Castellanos-Gomez A.; Van der Zant H. S. J. Atomically thin p–n junctions based on two-dimensional materials. Chem. Soc. Rev. 2018, 47, 3339–3358. 10.1039/C7CS00880E. 29683464

[ref28] Kunstmann J.; Mooshammer F.; Nagler P.; Chaves A.; Stein F.; Paradiso N.; Strucnk Ch.; Schuller Ch.; Seifert G.; Reichman D. R.; Korn T. Momentum-space indirect interlayer excitons in transition-metal dichalcogenide van der Waals heterostructures. Nature Phys. 2018, 14, 801–805. 10.1038/s41567-018-0123-y.

[ref29] Bosnick K. Transport in carbon nanotube p-i-n diodes. Appl. Phys. Lett. 2006, 89, 163121 10.1063/1.2360895.

[ref30] Jauregui L. A.; Joe A. Y.; Pistunova K.; Wild D. S.; High A. A.; Zhou Y.; Scuri G.; De Greve K.; Sushko A.; Yu C.-H.; Taniguchi T.; Watanabe K.; Needleman D. J.; Lukin M. D.; Park H.; Kim P. Electrical control of interlayer exciton dynamics in atomically thin heterostructures. Science 2019, 366 (6467), 870–875. 10.1126/science.aaw4194. 31727834

[ref31] Peimyoo N.; Deilmann T.; Withers F.; Escolar J.; Nutting D.; Taniguchi T.; Watanabe K.; Taghizadeh A.; Craciun M. F.; Thygesen K. S.; Russo S. Electrical tuning of optically active interlayer excitons in bilayer MoS2. Nat. Nano. 2021, 16, 888–893. 10.1038/s41565-021-00916-1. 34083771

[ref32] Chernikov A.; Berkelbach T. C.; Hill H. M.; Rigosi A.; Li Y.; Aslan O. B.; Reichman D. R.; Hybertsen M. S.; Heinz T. F. Exciton binding energy and nonhydrogenic rydberg series in monolayer WS_2_. Phys. Rev. Lett. 2014, 113, 076802 10.1103/PhysRevLett.113.076802. 25170725

[ref33] Wang G.; Chernikov A.; Glazov M. M.; Heinz T. F.; Marie X.; Amand T.; Urbaszek B. Colloquium: Excitons in atomically thin transition metal dichalcogenides. Rev. Mod. Phys. 2018, 90, 021001 10.1103/RevModPhys.90.021001.

[ref34] Mueller T.; Malic E. Exciton physics and device application of two-dimensional transition metal dichalcogenide semiconductors. npj 2D Mater. Appl. 2018, 2, 29 10.1038/s41699-018-0074-2.

[ref35] Cassabois G.; Valvin P.; Gil B. Hexagonal boron nitride is an indirect bandgap semiconductor. Nat. Photonics 2016, 10, 262 10.1038/nphoton.2015.277.

[ref36] Cannuccia E.; Monserrat B.; Attaccalite C. Theory of phonon-assisted luminescence in solids: application to hexagonal boron nitride. Phys. Rev. B 2019, 99, 081109 10.1103/PhysRevB.99.081109.

[ref37] Paleari F.; Miranda H. P. C.; Molina-Sánchez A.; Wirtz L. Exciton-phonon coupling in the ultraviolet absorption and emission spectra of bulk hexagonal boron nitride. Phys. Rev. Lett. 2019, 122, 187401 10.1103/PhysRevLett.122.187401. 31144865

[ref38] Chen H.-Y.; Sangalli D.; Bernardi M. Exciton-phonon interaction and relaxation times from first principles. Phys. Rev. Lett. 2020, 125, 107401 10.1103/PhysRevLett.125.107401. 32955294

[ref39] Jin C.; Kim J.; Suh J.; Shi Z.; Chen B.; Fan X.; Kam M.; Watanabe K.; Taniguchi T.; Tongay S.; Zettl A.; Wu J.; Wang F. Interlayer electron-phonon coupling in WSe2/hBN heterostructures. Nat. Phys. 2017, 13, 127 10.1038/nphys3928.

[ref40] Chow C.; Yu H.; Jones A.; Schaibley J.; Koehler M.; Mandrus D.; Merlin R.; Yao W.; Xu X. Phonon-assisted oscillatory exciton dynamics in monolayer MoSe_2_. npj 2D Materials and Applications 2017, 1, 33 10.1038/s41699-017-0035-1.

[ref41] Shree S.; Semina M.; Robert C.; Amand T.; Balocchi A.; Manca M.; Courtade E.; Marie X.; Taniguchi T.; Watanabe K.; Glazov M.; Urbaszek B. Observation of exciton-phonon coupling in MoSe_2_ monolayers. Phys. Rev. B 2018, 98, 035302 10.1103/PhysRevB.98.035302.

[ref42] Lin K.-Q.; Ong C.; Bange S.; Faria Junior P.; Peng B.; Ziegler J.; Zipfel J.; Bauml C.; Paradiso N.; Watanabe K.; Taniguchi T.; Strunk C.; Monserrat B.; Fabian J.; Chernikov A.; Qiu D.; Louie S.; Lupton J. Narrow-band high-lying excitons with negative-mass electrons in monolayer WSe_2_. Nat. Commun. 2021, 12, 5500 10.1038/s41467-021-25499-2. 34535654 PMC8448890

[ref43] Funk V.; Wagner K.; Wietek E.; Ziegler J.; Forste J.; Lindlau J.; Forg M.; Watanabe K.; Taniguchi T.; Chernikiv A.; Hogele A. Spectral asymmetry of phonon sideband luminescence in monolayer and bilayer WSe_2_. Physical Review Research 2021, 3, L042019 10.1103/PhysRevResearch.3.L042019.

[ref44] Reichardt S.; Wirtz L. Nonadiabatic exciton-phonon coupling in Raman spectroscopy of layered materials. Science Adv. 2020, 6, eabb5915 10.1126/sciadv.abb5915. PMC7413722 32821840

[ref45] Stern H. L.; Cheminal A.; Yost S. R.; Broch K.; Bayliss S. L.; Chen K.; Tabachnyk M.; Thorley K.; Greenham N.; Hodgkiss J. M.; Anthony J.; Head-Gordon M.; Musser A. J.; Rao A.; Friend R. H. Vibronically coherent ultrafast triplet-pair formation and subsequent thermally activated dissociation control efficient endothermic singlet fission. Nat. Chem. 2017, 9, 1205–1212. 10.1038/nchem.2856. 29168494

[ref46] Bian Q.; Ma F.; Chen S.; Wei Q.; Su X.; Buyanova I. A.; Chen W. M.; Ponseca C. S. Jr; Linares M.; Karki K. J.; Yartsev A.; Inganäs O. Vibronic coherence contributes to photocurrent generation in organic semiconductor heterojunction diodes. Nat. Commun. 2020, 11, 617 10.1038/s41467-020-14476-w. 32001688 PMC6992633

[ref47] Novoderezhkin V. I.; Romero E.; Prior J.; van Grondelle R. Exciton-vibrational resonance and dynamics of charge separation in the photosystem II reaction center. Phys. Chem. Chem. Phys. 2017, 19, 5195–5208. 10.1039/C6CP07308E. 28149991

[ref48] Romero E.; Auguli R.; Novoderezhkin V. I.; Faretti M.; Thieme J.; Zigmantas D.; van Grondelle R. Quantum coherence in photosynthesis for efficient solar-energy conversion. Nat. Phys. 2014, 10, 676–682. 10.1038/nphys3017. 26870153 PMC4746732

[ref49] Romero E.; Novoderezhkin V. I.; van Grondelle R. Quantum design of photosynthesis for bio-inspired solar energy conversion. Nature 2017, 543, 355–365. 10.1038/nature22012. 28300093

[ref50] Arp T. B. qmolabucr/wse2mose2: Publication Release, version v1.2; Zenodo, 2021; 10.5281/zenodo.5516680.

[ref51] Barati F. Optoelectronics investigations of electron dynamics in 2D-TMD semiconductor heterostructure photocells: from electron-hole pair multiplication to phonon assisted anti-Stokes absorption. Thesis, University of California, Riverside, CA, 2018.

[ref52] Arp T. B.; Gabor N. M. Multiple parameter dynamic photoresponse microscopy for data-intensive optoelectronic measurements of van der Waals heterostructures. Rev. Sci. Instrum. 2019, 90, 023702 10.1063/1.5085007. 30831738

[ref53] Kresse G.; Furthmüller J. Efficient iterative schemes for ab initio total-energy calculations using a plane-wave basis set. Phys. Rev. B 1996, 54, 11169 10.1103/PhysRevB.54.11169. 9984901

[ref54] Kresse G.; Hafner J. Ab initio molecular dynamics for liquid metals. Phys. Rev. B 1993, 47, 558 10.1103/PhysRevB.47.558. 10004490

[ref55] Kresse G.; Furthmüller J. Efficiency of ab-initio total energy calculations for metals and semiconductors using a plane-wave basis set. Comput. Mater. Sci. 1996, 6, 15 10.1016/0927-0256(96)00008-0. 9984901

[ref56] Blöchl P. E. Projector augmented-wave method. Phys. Rev. B 1994, 50, 17953 10.1103/PhysRevB.50.17953. 9976227

[ref57] Perdew J. P.; Chevary J. A.; Vosko S. H.; Jackson K. A.; Pederson M. R.; Singh D. J.; Fiolhais C. Atoms, molecules, solids, and surfaces: Applications of the generalized gradient approximation for exchange and correlation. Phys. Rev. B 1992, 46, 6671 10.1103/PhysRevB.46.6671. 10002368

[ref58] Wang Y.; Perdew J. P. Correlation hole of the spin-polarized electron gas, with exact small-wave-vector and high-density scaling. Phys. Rev. B 1991, 44, 13298 10.1103/PhysRevB.44.13298. 9999531

[ref59] Kresse G.; Joubert D. From ultrasoft pseudopotentials to the projector augmented-wave method. Phys. Rev. B 1999, 59, 1758 10.1103/PhysRevB.59.1758.

[ref60] Grimme S. Semiempirical gga-type density functional constructed with a long-range dispersion correction. J. Comput. Chem. 2006, 27, 1787 10.1002/jcc.20495. 16955487

[ref61] Togo A.; Tanaka I. First principles phonon calculations in materials science. Scripta Mater. 2015, 108, 1 10.1016/j.scriptamat.2015.07.021.

[ref62] Grosso G.; Grosso; Parravicini G. P. Solid State Physics; Academic Press: London, 2000.

[ref63] Franck J. Elementary processes of photochemical reactions. Trans. Faraday Soc. 1926, 21, 536–542. 10.1039/tf9262100536.

[ref64] Condon E. U. A Theory of Intensity Distribution in Band Systems. Phys. Rev. 1926, 28, 1182 10.1103/PhysRev.28.1182.

