# Supplementary material for: Vibronic Exciton–Phonon States in Stack-Engineered van der Waals Heterojunction Photodiodes
Source: Nano Lett. 2022 Jul 5;22(14):5751–8. doi: 10.1021/acs.nanolett.2c00944 (PMC9335870; doi:10.1021/acs.nanolett.2c00944)
Supplement: Supplementary file 1 — nl2c00944_si_001.pdf [file nl2c00944_si_001.pdf]

Supplementary Materials for

Vibronic exciton-phonon states in stack-engineered van der Waals heterojunction photodiodes

Authors: Fatemeh Barati[1,2,†], Trevor B. Arp[1,2,†], Shanshan Su[3], Roger K. Lake[3], Vivek Aji[2], Rienk van Grondelle[4,5,*], Mark S. Rudner[6,7*], Justin C.W. Song[8,*], Nathaniel M. Gabor[1,2,5*]

Affiliations:

[1]Laboratory of Quantum Materials Optoelectronics, University of California, Riverside, CA 92521, USA.

[2]Department of Physics and Astronomy, University of California, Riverside, CA 92521, USA.

[3]Laboratory for Terahertz and Terascale Electronics (LATTE), Department of Electrical and Computer Engineering, University of California - Riverside, Riverside, California 92521, USA.

[4]Department of Physics and Astronomy, Faculty of Sciences, Vrije Universiteit Amsterdam, De Boelelaan 1081, 1081 HV, Amsterdam, Nederland.

[5]Canadian Institute for Advanced Research, MaRS Centre West Tower, 661 University Avenue, Toronto, Ontario ON M5G 1M1, Canada.

[6]Department of Physics, University of Washington, Seattle, WA 98195, USA

[7]Niels Bohr Institute, University of Copenhagen, 2200 Copenhagen, Denmark

[8]Division of Physics and Applied Physics, School of Physical and Mathematical Sciences, Nanyang Technological University, Singapore 637371.

*r.van.grondelle@vu.nl, rudner@nbi.ku.dk, justinsong@ntu.edu.sg, nathaniel.gabor@ucr.edu

† These authors contributed equally to this work

This PDF file includes:

Supplementary Text
Figs. S1.1 to S4.10
Including Citations to References 51-64

Section S1. $WSe_2/MoSe_2$ Device Fabrication

In this work, we present intensive spectroscopic, electronic, and optoelectronic characterization of precisely fabricated heterostructure devices composed of bilayer WSe_2 and monolayer $MoSe_2$. Based on our previous temperature dependent electronic transport study on the same material system[17,51], this unique combination of materials was chosen due to its indirect band gap, unique phonon band structure, and long-lived interlayer excitons expected at infrared wavelengths.

The heterostructure devices are assembled using a highly customized, temperature-controlled transfer microscope that ensures the interface between the two layers has no intentional contact to polymer films. The first step of the fabrication is to make the back-gate assembly as shown in Fig. S1b. The back gate consists of a layer of graphite (2 nm thick) as the conductor and a hexagonal boron nitride (h-BN) layer (12 nm) on top as the gate dielectric. The graphite layer is picked up using the h-BN and mounted on the prefabricated gate contacts. Fig. S1a shows the device geometry with all components in two different perspectives. Fig. S1b shows optical images of the three stages of the fabrication process from left to right. The left image in Fig. S1b shows the first step of the fabrication which is the gate assembly on the SiO_2 substrate. The next step is to write source-drain contacts on top of the bottom h-BN layer, these layers are labeled as $MoSe_2$ contact and WSe_2 contact shown in Fig. S1b middle. These contacts provide electrical access to each layer of the stack. For device 1 the $MoSe_2/WSe_2$ heterostructure was fabricated using mechanical exfoliation of WSe_2 and $MoSe_2$ flakes from bulk crystal (2D semiconductors) onto Si wafers coated with 290-nm-thick SiO_2. The silicon substrate is implemented as a back gate to tune the carrier density at the interface. Electrical access to the two layers of $MoSe_2$ and WSe_2 is achieved through independent electrical contacts to each material. The details of the fabrication process can be found in our previous study[17].

To minimize interfacial contamination, the two layers of the stack (WSe_2 and $MoSe_2$) are picked up via the top h-BN layer one after another in a two-step transferring process and mounted on the source drain contacts on the prefabricated gate. To get rid of possible impurities and destructive bubbles formed in between the layers of the heterostructure the device is finally annealed at 250 °C under Ar gas for one hour. The final step is to mount the device into a chip carrier within the measurement setup. The chip carrier is a patterned substrate of sapphire with gold for electrical access to the device. The electronic contacts on the SiO_2 substrate are wire

bonded into the conductive pads of the chip carrier shown in Fig. S1 (left). The device is then soldered to the electronic pads of the cryostat and silver painted for further electronic experiments shown in Fig. S1c (right).

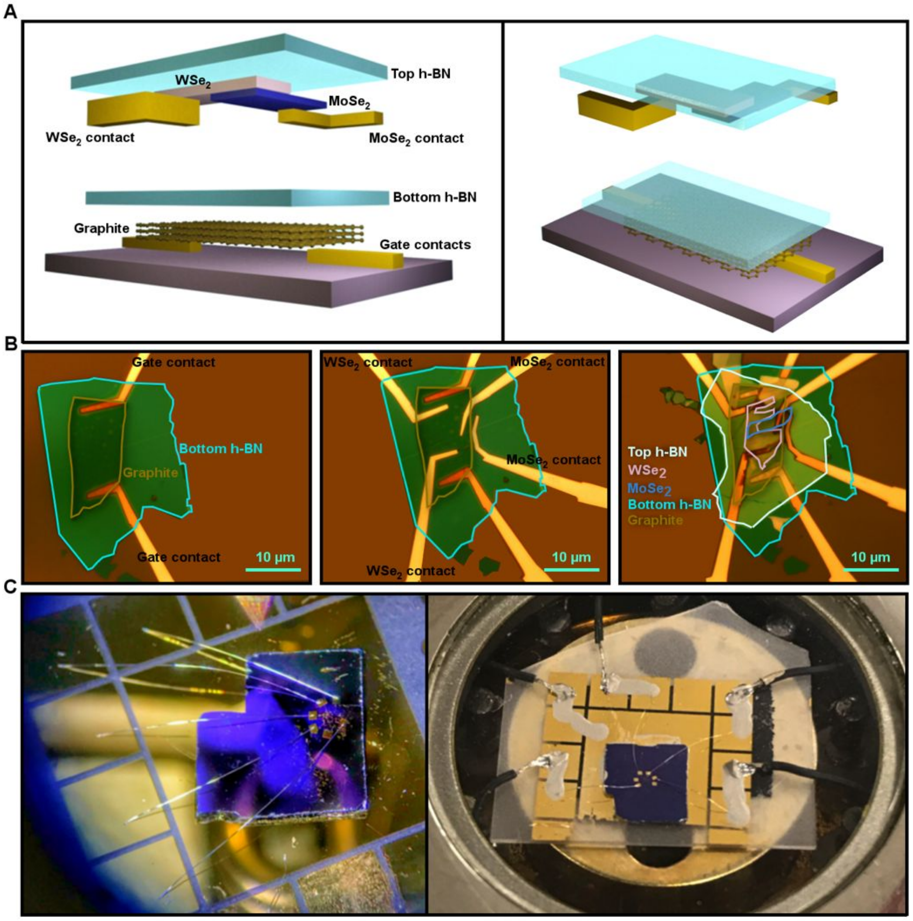

Fig. S1.1 a, Two different perspectives of the device geometry, components labeled. **b,** Optical microscope images of the device showing the three-step fabrication process. Back-gate assembly consisting of electrical contacts made of gold, a layer of graphite and a layer of h-BN respectively from bottom to top. (left). Source-drain contacts are then drawn on top of the bottom h-BN layer (middle) for electrical access to the device. A stack of h-BN/WSe_2/$MoSe_2$ is then laminated onto these prefabricated electrical contacts (right). **c**, Photographs of the devices wire-bonded into chip carriers. Device 2 (left) was contacted using a probe station cryostat (see Section S2.2). Device 1 (right) was contacted by soldering chip carrier to electrical pins of the cryostat (see section S2.1).

Section S2.1. The Supercontinuum Scanning Photocurrent Spectroscopy Microscope

The supercontinuum scanning photocurrent spectroscopy microscope (SSPSM) is a powerful tool designed to explore quantum optoelectronic properties of atomic scale devices by integrating optics and electronics into a combined quantum transport microscope. The SSPSM is equipped with a helium flow optical cryostat that enables both electrical access and optical illumination to nanoscale devices through electrical feedthroughs and a transparent window. This cryostat provides a stable and controllable low-pressure environment via a turbo pump system and can be cooled down to temperatures of $T = 4$ K.

The SSPSM microscope employs a supercontinuum Fianuim white laser as the light source along with Princeton Instrument monochromator for spatially and spectrally resolved supercontinuum excitations. The light source we use for this microscope is a Fianium WhiteLaseTM SC400-8; a picosecond pulsed laser pump (repetition rate = 20 MHz, pulse width = 6 ps) is coupled into a micro-structured optical fiber (the non-linear medium) of 1.5-meter length. At the output of the fiber, the collimated white beam of light has an average power of 8 W and a power spectral density of 4 mW/nm that ranges from λ~400-2200 nm. Fig. S2.1 illustrates a schematic diagram of the microscope. The light that reaches the monochromator through the left optical line can be used for spatially and spectrally resolved laser excitations. The monochromatic laser light then generates a diffraction limited beam spot by passing through several optical components[55].

To probe the optoelectronic response of the samples we use scanning beam photoexcitation, as shown in Fig. S2.1 (GVS, L7 and L8) to generate a collimated beam of light on the back aperture of an objective. As the angle of the collimated beam is shifted the resulting diffraction limited beam spot that is scanned over the nanoscale device. The resulting current is amplified using a sub-100 fA resolution pre-amplifier over long averaging times (10-1000 ms) at low optical power. The reflected light from the sample transmits through the BS and reflects to a lens (L9) and gets collected by a photodiode detector (PD). The photodiode converts the optical power to electrical power as a photovoltage; the intensity of the photovoltage is monitored to form a simultaneous reflection image of the sample. To monitor and calibrate the output of the light source over the whole wavelength range, we use two photodetectors. A Si switchable gain detector (Thorlabs: PDA36A) is sensitive to the light from UV to NIR wavelength range (350-1100 nm),

and an InGaAs photodetector (Thorlabs: DET10C) on the other hand is designed to detect light signals ranging from 900 to 1700 nm.

To achieve the highest performance of this microscope over the broad spectral range (400-2200 nm), optical components must match the wavelength range that we choose to conduct experiments. There are three different beam splitter coatings to cover the whole wavelength range. Thorlabs non- polarizing achromatic beam splitter cubes can provide a uniform reflection in these three ranges: BS028 for Vis (400-700 nm), BS029 for NIR (700-1100 nm), and BS030 for IR (1000-1600 nm). The beam splitters used for this microscope reflect 90% of the light to the sample; the 10% of the light that transmits through the BS can get detected with a power sensor (PS) coupled to a power meter (PM). The power sensor response function should also match the wavelength range of the microscope; using two power sensors (Thorlabs: S130C, and S132C) we can accurately detect the optical power in the ranges of 400-1100 nm and 700-1800 nm respectively in the power range of 500 pW-500 mW.

Data, in the form of two-dimensional maps of photocurrent and reflection, is acquired and displayed using python based custom control software[52]. Using the SSPSM microscope, we can perform spatial imaging as a function of wavelength by fixing the spectrum at a specific wavelength and conducting 2D spatial photocurrent mappings over the surface of the sample, while changing other parameters such as V_{SD}, V_G, or temperature in order to make 3D or 4D data cubes. We can also perform spectral imaging with a narrow resolution of 1 nm by fixing the laser beam at a specific spot on the device (e.g., the heterostructure as identified by correlating scanned reflection images to know device geometries) while tuning for example, the excitation wavelength. Additional parameters such as V_{SD}, V_G, or temperature are then tuned to form a multi-dimensional data set.

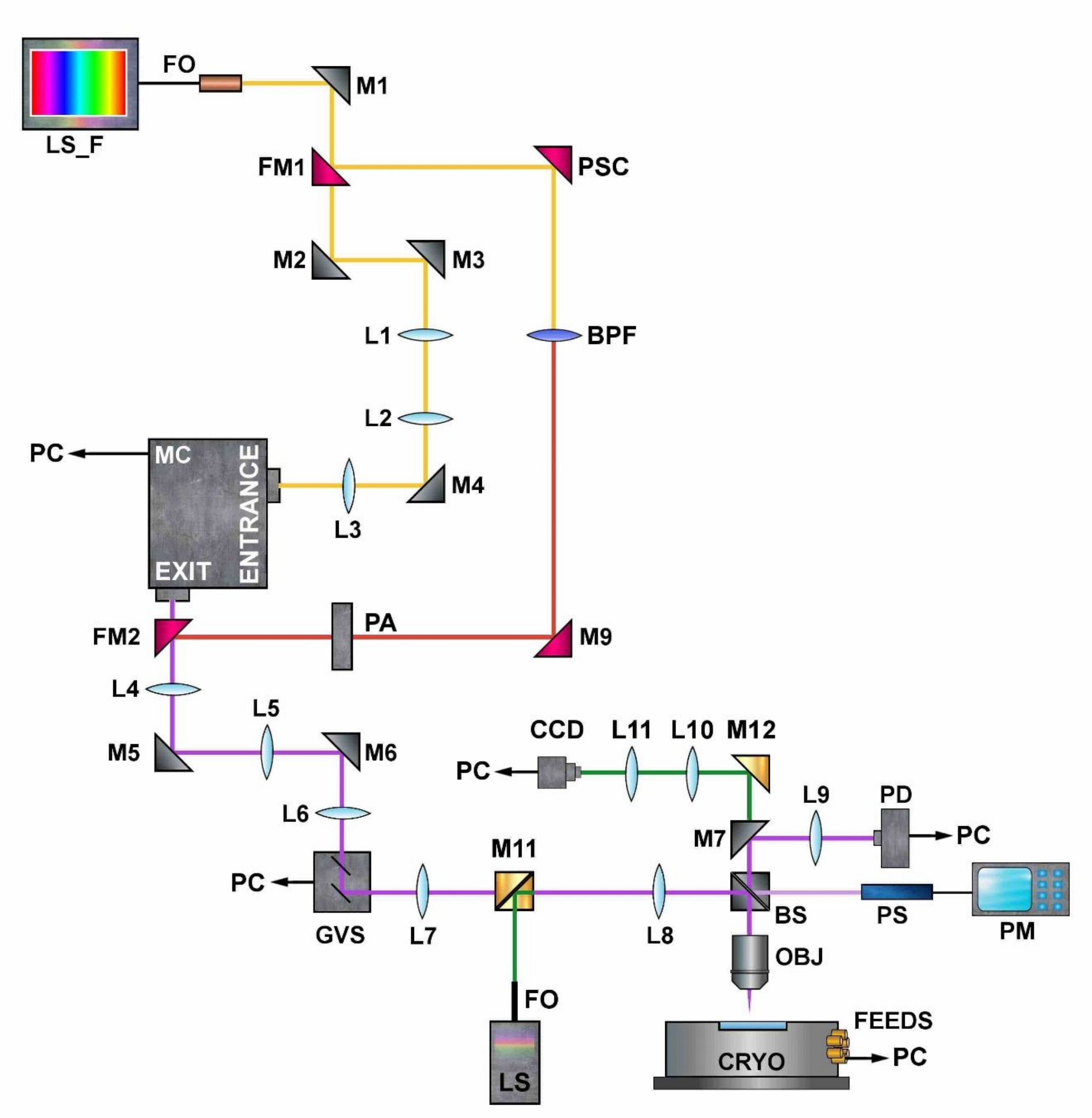

Fig. S2.1 Schematic diagram of the supercontinuum scanning photocurrent spectroscopy microscope (SSPSM). Constituent components of the microscope comprise: light source of Fianium (LS_F), FO (fiber optics), monochromator (MC), mirrors (M), lenses (L), flip mount mirrors (FM), galvos system (GVS), beam splitter (BS), objective (OBJ), power sensor (PS), power meter (PM), photodiode detector (PD), CCD camera (CCD), cryostat (CRYO), PSC (periscope), band pass filter (BPF). Electronic experiments are conducted through the optical CRYO, while optical illumination is introduced via OBJ to devices[51].

Section S2.2. Multi-Parameter Dynamic Photoresponse Microscopy Measurement Details

Data intensive imaging of the sample phase space was performed using the Multi-Parameter Dynamic Photoresponse Microscopy (MPDPM) technique described previously by Arp & Gabor. Below we give a basic description of the most relevant aspects of the optics to this experiment, but more details on the hardware and methodology can be found in reference[52]. Data shown in the main figures is condensed from the MPDPM data analysis process, while the full imaging data sets can be found in section S4.3.

The core of the optical system is a Coherent MIRA 900 OPO ultrafast pulsed laser which generates 150 fs pulses with controllable wavelength from 1150 nm to 1550 nm at a 75 MHz repetition rate. The output of the laser is attenuated to control the laser power and fed into a scanning beam system similar to GVS, L7 and L8 in Fig. S2.1. After the second lens (L8 in Fig. S2.1) the MPDPM optics differ significantly from the SSPSM setup in order to get a stronger optical probe. In MPDPM a strong and highly local optical probe is required for best results, therefore the output of the scanning optics is directed into a GRIN (Gradient Index of Refraction) lens, which performs a similar function to the objective lens but with dramatically lower pulse dispersion. Combined with the fact that the OPO laser output has significantly higher power than the output of the SSPSM monochromator (order of mW at the sample) the pulse fluence is several orders of magnitude larger in the MPDPM setup than the SSPSM.

Photocurrent was measured using a lock-in amplifier. The laser beam was modulated by an optical chopper and the signal was passed through a pre-amplifier then locked-in to the chopper frequency. This was necessary to remove the substantial dark current that occurs due to the sample diode characteristics (see Fig. 1f). The data was then acquired through a National Instruments PCIe-6323 Data Acquisition Card. In addition to the photocurrent signal, the reflected light from the sample was continuously acquired along with the laser power.

The imaging data set shown in Fig S4.3, along with several similar image sets, was acquired using the "datacube" method from MPDPM, i.e. automatically taking a series of 2D scanning images as a function of one variable (with all other variables held constant) to form a three-dimensional data set, and then taking a series of these datacubes incremented over variable to get a 4-dimensional data set. Each set involved hundreds to thousands of images measured fully automatically over the course of several hours to a few days. For these longer measurements the main concern is laser stability, as any variation in the optics will cause the image location to drift.

To counter this, careful attention was paid to the alignment of the optics to avoid any geometrical aberration and the laser was run continuously for several days prior to any long imaging measurement to stabilize its performance. When the system is stable the main source of drift is thermal expansion of the various optical components; therefore, temperature and airflow of the lab is carefully maintained during measurements.

Even with the extra considerations for stability there will inevitably be some drift in the images, which must be corrected for in the data. There are multiple options but given that the laser power was monitored and shown to be constant throughout the measurement and the drift is small, the best method was to re-orient the data based on the reflection image. To find the correction for a given reflection image we use an algorithm that shifts the image by an integer number of pixels and calculates the difference between that image and the first image in the data set. By brute force the algorithm determines the shift that produces the minimum difference and uses that as the correction. This allows for correction that is accurate to the pixel resolution. For computational simplicity (and because the drift within datacubes was sub-pixel) we perform this procedure by comparing the first image in each 3D datacube to determine the shift for the whole cube.

Fig. S2.2 shows the drift correction over time for the longest measurement taken on this sample, which spanned 53 hours. In Fig. S2.2a we see the first and last images in the data set, and then the last image with the correction applied. Applying the correction removes some information near the edges, however the images were taken with a significant margin around the active area of the sample to prevent the drift correction from removing meaningful information. Fig. S2.2b shows the drift correction as a function of time over the 53-hour span of the measurement. Critically, we see that the drift undergoes a random walk, as we would expect from thermally dominated drift, and that over the course of 53 hours it only shifts by about 3 microns (approximately 10 pixels), an acceptable level of drift that can be corrected for without harm to the data.

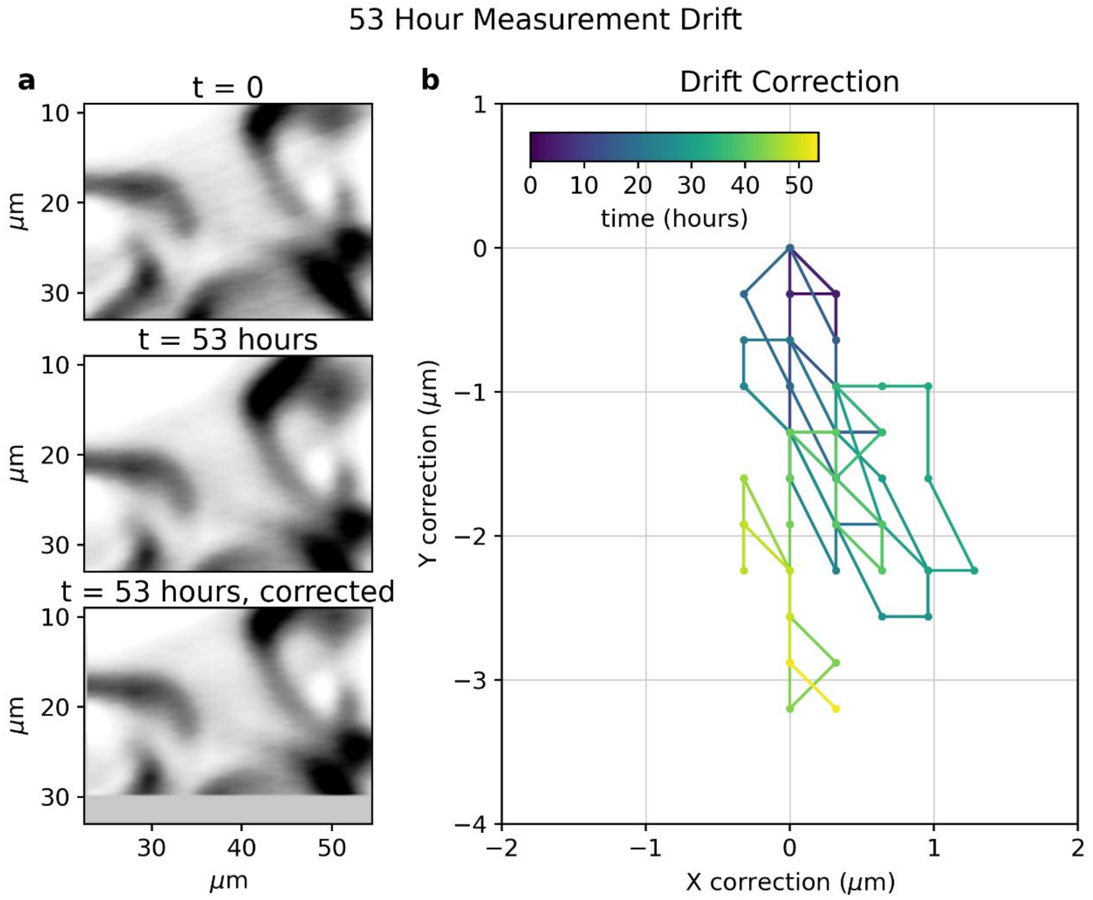

Fig. S2.2 Drift correction for a 53-hour measurement imaging measurement. **a**, shows the reflection image at the start of the measurement (top) and the end of the measurement (middle) and then the last image corrected to be spatially consistent with the first image (bottom). **b**, the required drift correction as a function of time over the measurement.

S3. Phonons of the heterostructure

S3.1 Evidence for Phononic Origin of the Sidebands

In the WSe_2-$MoSe_2$ heterojunction devices, we establish that photocurrent peaks arise from absorption resonances due to photoexcited interlayer excitons with dipole moments $\vec{p}$ that are constrained to point out-of-plane. For both of these interlayer excitons, the observed energy spacing $\Delta\varepsilon$ = 30 meV is *similar* as confirmed both by Lorentzian fitting (see Fig. 2 in the main text, according to convention in the PL community) and by the Fourier transformed data. In contrast, similar periodic sideband behavior is not observed in the photocurrent due to intralayer excitons (See Supplementary Figure S4.1 and surrounding discussion below).

As a key point, the energy spacing does not seem to be sensitive to the distinct microscopic structure of the two types of interlayer excitons ($K \rightarrow K$ vs. $\Gamma \rightarrow K$), and it is therefore highly unlikely that such periodic structure arises from random defect states below each of the exciton absorption (owing to the strong coincidence of energies within the electronic structure of *two distinct interlayer excitons*). The similarity in both excitons' spectral fingerprints indicates that periodic photocurrent peaks arise from a similar origin. To observe such sidebands due to potential modulation would require very careful fine tuning of the potential. We note that the side bands occur for a broad range of gate voltages, making fine-tuning effects unlikely.

S3.2 Phonon calculation details

In this Section, we investigate numerically the vibrational modes in the heterostructure in an effort to identify the candidate modes that can be involved in the vibronic-like photoresponse. We note that this numerical study treats the electronic system in its insulating ground state (without excitons present) in order to identify phonon modes whose vibrational energies are consistent with the observed vibronic-like behavior. Vibrational coupling to the interlayer excitons at such large vibrational energies (~30 meV) suggests that the relevant vibrational modes are optical phonon modes with flat band structure on the scale of the thermal energy $k_b T$. Using detailed phonon dispersion calculations (described below) we identify such phonon modes (Einstein modes) within the $MoSe_2$/WSe_2 heterostructure and explore the detailed properties of these modes. We find that the relevant modes exhibit flat bands with excitation energies near 30 meV.

The phonon dispersion of the heterostructure $MoSe_2$/WSe_2 with spin-orbit coupling (SOC) was calculated using the Vienna ab initio simulation package (VASP)[53-55] in the projected-

augmented-wave method[56]. The generalized gradient approximation (GGA) with Perdew-Burke-Ernzerhof (PBE) parametrization[57-59] was used for the exchange correlation energy. The Van der Waals interactions between the layers are accounted by using the DFT-D2 Grimme's method[60]. The kinetic energy cutoff for our calculation is set as 500 eV.

For all structural relaxations, the convergence tolerance on the Hellmann-Feynman forces is less than 10^{-7} eV/Å. An 8×8×1 Γ-centered Monkhorst-Pack k-point mesh is used for 2D films. The vacuum layer added to the system is nearly 20 Å. Under these settings, the lattice constant of $MoSe_2/WSe_2$ is 3.3259 Å. The lattice mismatch between $MoSe_2$ and WSe_2 is less than 1%. Both of the two materials contain heavy elements; therefore, SOC is included for all calculations. The phonon dispersion is calculated for a 4×4×1 supercell using Phonopy[61].

S3.3 Phonon dispersion relation and density of states

We first examine the phonon modes in the region near 30 meV. As previously reported, there are a variety of Raman active and Raman inactive phonon modes near that energy[62]. To proceed we use the DFT calculations discussed in section S3.1 to model the dispersion of the phonon modes, and their density of states, shown in Fig. S3.1. Here, the faded blue bar marks 30 meV, but is broadened slightly to represent the range of energies that would be consistent with the 30 meV mode observed in the data at T = 20 K. From Fig. S3.1, we see that there are multiple strongly active modes with bands near 30 meV. Each of the 27 bands in the dispersion corresponds to a particular mode of atomic vibration in the $WSe_2/MoSe_2$ heterostructure. Using the detailed results of our calculations, we next explore the atomic motions and band dispersions of each of these modes and identify which are potentially relevant to the absorption within the heterostructure. The right side of Fig. S3.1 shows the phonon density of states (DOS) versus phonon energy for the heterostructure, shown in black, which is sum of the DOS of the WSe_2 and $MoSe_2$ layers in blue and green respectively.

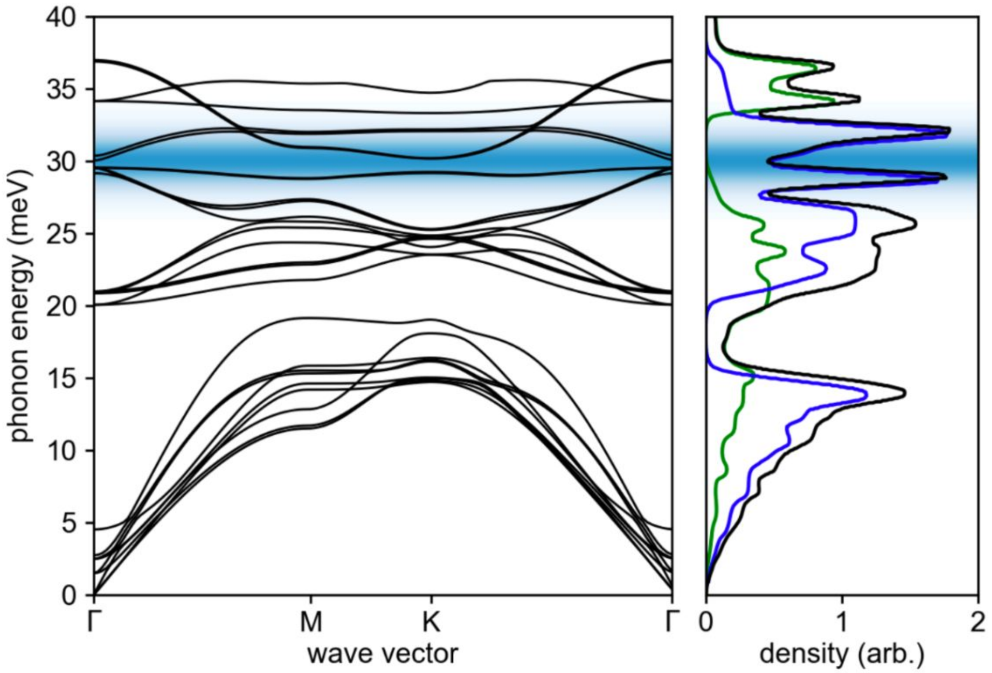

Fig. S3.1 Dispersion of DFT calculated phonon modes (left), and, phonon density of states, for the $MoSe_2$ (green line), WSe_2 (blue line) and heterostructure (black line). Blue bar shows 30 meV with thermal broadening of k_bT for $T = 20$ K (right).

Fig. S3.2 shows the displacements for the 7 modes at Γ near 30 meV corresponding to the 7 optical phonon branches shown in Fig. S3.3. The phonons mainly consist of in-plane vibrations, (E_{2g} modes) with some out-of-plane vibrations (A_{1g} modes). This indicates that there are several optical phonons near the energy of the oscillations we observe.

By comparing to our data, we identify candidate phonon modes that may couple strongly to an out-of-plane dipole. In Fig. 2, we see sharp peaks at 30 meV for photocurrent spectra involving transitions at different locations in the Brillion zone ($K \rightarrow K$ and $\Gamma \rightarrow K$). A vibrational (phonon) mode responsible for such oscillations must have an excitation energy of approximately 30 meV at both the K and Γ points. In Fig. S3.3 we plot the seven phonon modes that are near 30 meV at the Γ point. These bands correspond to the phonon motion schematically depicted in Figure S3.2. Having energies close to 30 meV, they can all in principle contribute to the phonon sidebands we observe in the main text.

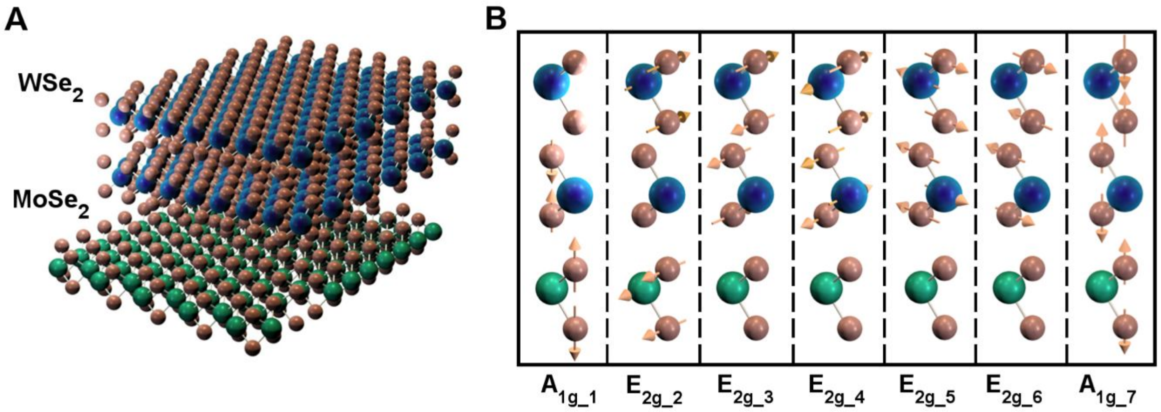

Fig. S3.2 **a**, Device atomic structure geometry. **b**, Visualization of optical phonons in the heterostructure along with their spectroscopy classification.

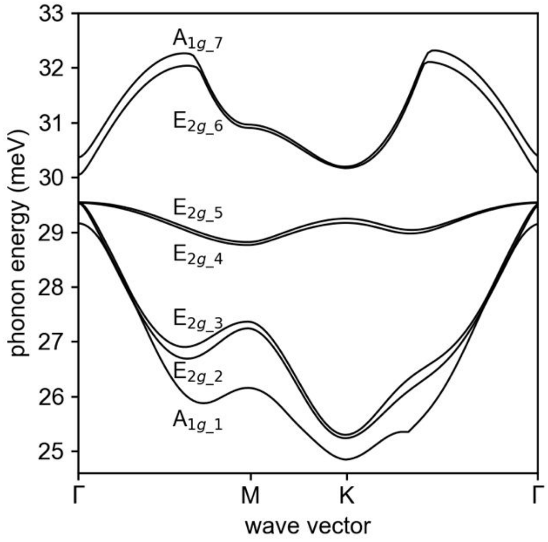

Fig. S3.3 DFT calculated phonon dispersion for modes with Γ-point energies near 30 meV.

S4. Additional Data Analysis: Photocurrent Spectroscopy

S4.1. Photocurrent Spectroscopy of Intralayer Excitons

In this section we compare the photocurrent response of *intralayer* excitons to that of interlayer excitons within the vdW heterojunction. We show that the intralayer exciton photocurrent exhibits several key differences when compared to the interlayer photocurrent: First, photocurrent due to intralayer excitons *does not* exhibit regularly spaced peaks like those shown for interlayer excitons as in Fig. 2. Second, photocurrent due to intralayer excitons does not require the device to be tuned to charge neutrality and exhibits gate voltage dependence for positively and negatively charged trion species, consistent with ordinary behavior. We observe features that originate in both the $MoSe_2$ and WSe_2, with photocurrent excitation energies that match the relevant photoluminescence energies, consistent with ordinary photoresponse of intralayer excitons.

With high spectral resolution we tune the excitation energy close to the heterostructure peaks observed in the photoluminescence (Fig. 1b) and measure interlayer photocurrent. Fig. S4.1a shows photocurrent as a function of photon excitation energy from 1.55 eV to 1.37 eV (wavelength range between 800-900 nm), and V_G between -20 to 20 V. We observed the appearance of three peaks at different photon energies that shift with gate voltage from V_G = -20 V to 20 V. Notably, no sidebands are observed in the photoresponse of these peaks.

Fig. S4.1b shows the position of the peaks versus photon energy. We observed a peak at 1.53 eV, which we attribute to the neutral exciton (X_{Mo}^0) in $MoSe_2$. This peak does not shift as a function of gate voltage and corresponds approximately with the sharpest peak observed in the PL spectrum of $MoSe_2$, Fig. 1b. We attribute the photocurrent peak observed at 1.49 eV to the positively charged exciton (trion) of $MoSe_2$ (labeled X_{Mo}^+), which redshifts over the gate voltage range with a small slope, consistent with ordinary trion behavior. The photocurrent peak observed at 1.443 eV is attributed to the positive exciton (trion) X_W^+ originating from WSe_2. This peak again redshifts gradually as a function of gate voltage and corresponds closely to the PL peak position observed for WSe_2. We note that none of these peaks are observed exclusively at charge neutrality, instead exhibiting gate voltage dependence consistent with ordinary behavior.

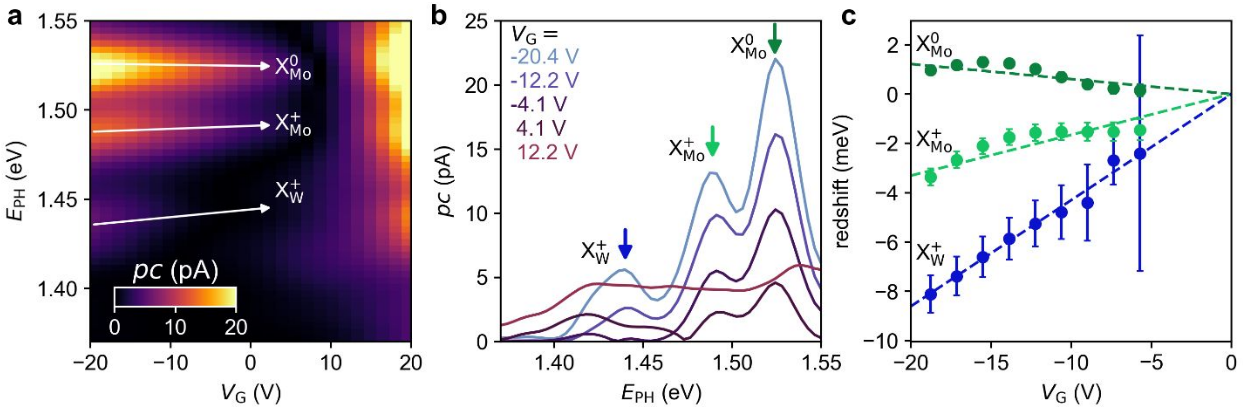

Fig. S4.1 Gate voltage dependence of the excitation photon energy close to the energy of the intralayer excitons at room temperature. **a**, Color map of photocurrent (dark current removed, see Section S4.2.) versus excitation energy (1.37 eV to 1.55 eV) and V_G at a fixed V_{SD} = 6 V (T = 300 K). **b**, Line cuts of interlayer photocurrent vs. excitation photon energy at several values of V_G showing the existence of three peaks, the neutral exciton peak in $MoSe_2$ at 1.52 eV, the positive exciton peak in $MoSe_2$ at 1.49 eV, and the positive exciton peak in WSe_2 at 1.44 eV. **c**, Red shift of the peak positions versus gate voltage, neutral exciton in $MoSe_2$ (in dark green), positive exciton peak in $MoSe_2$ (in medium green), and positive exciton peak of WSe_2 (in blue). Peak positions calculated by fitting the photocurrent at constant V_G to a function of three Lorentzian peaks, error bars indicate uncertainty in the fit. Dashed lines show a linear fit to the redshift extrapolated to V_G = 0 V.

S4.2 Low Temperature Photocurrent Spectroscopy in the Vicinity of Charge Neutrality

The low temperature photocurrent spectroscopy data shown in Fig. 2 was measured using the SSPSM setup described in section S2.1 on device 1 in an optical cryostat cooled with liquid helium to approximately 20 Kelvin. The spectral resolution of E_{PH} is limited by a tradeoff in the SSPSM monochromator where increasing the spectral resolution decreases the pulse power (or equivalently, the signal to noise).

In the low temperature measurements, the dominant current signal is the dark current of the device with respect to V_G. Fig. S4.2a shows the raw current for the measurement shown in Fig. 2e (the Fig. 2a measurement is similar). We see that the raw signal is an order of magnitude larger than the photocurrent scale shown in Fig. 2e due to a large dark current. Fig. S4.2b shows that the photocurrent, i.e. the current varying with $E_{PH,}$ is a small modulation on top of the dark current. There are several ways to process and remove the dark current, we use the simplest method, which

is also advantageous for analyzing the photocurrent oscillations, that is we base our analysis on the derivatives with respect to E_{PH}, where the constant dark current drops out. For the purposes of displaying the data in Fig. 2a,e (and Fig. S4.3a,e) we set the minimum of the color scale at some threshold (shown as the grey dashed line in Fig. S4.2b) that displays the photocurrent in the most active region of V_G. This is purely visual, to give contrast to the photocurrent oscillations, and does not affect the subsequent analysis.

Fig. S4.3 breaks down the low temperature spectroscopy data in detail, showing line cuts of the data and the first and second derivatives of photocurrent with respect to E_{PH} to visually display the photocurrent oscillations. The second derivative data in Fig. S4.3c,f was Fourier transformed to generate Fig. 2c,f.

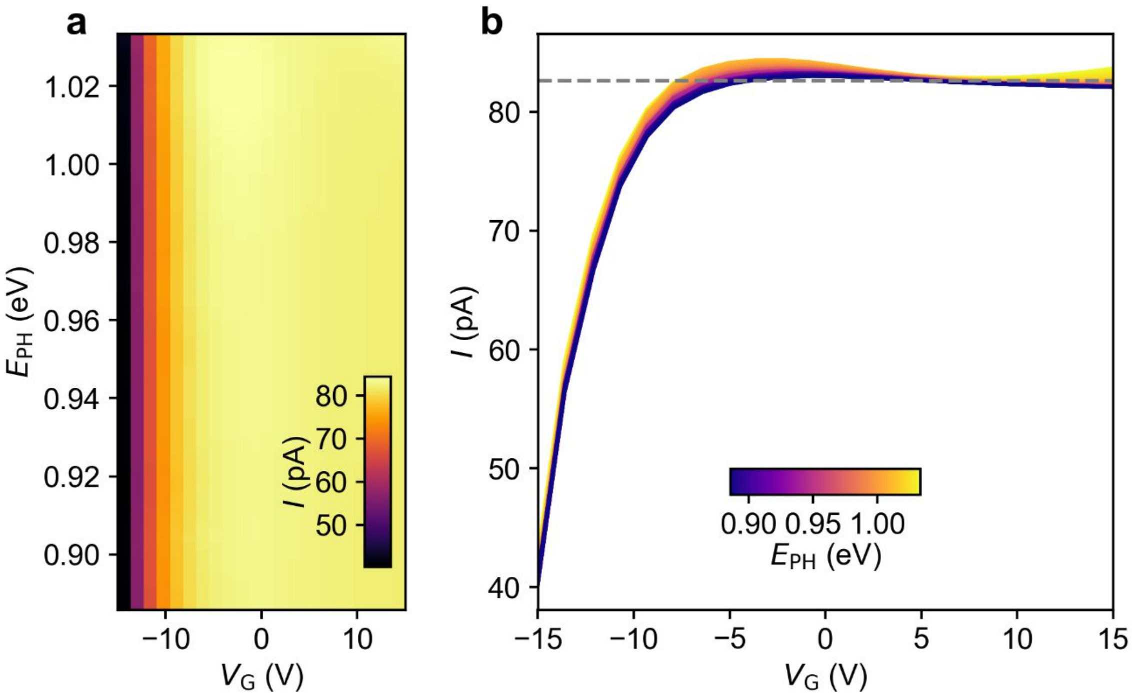

Fig. S4.2 Raw low temperature photocurrent data. **a**, Raw Photocurrent, I versus E_{PH} and V_G, showing the primary effect from dark current. **b**, Raw photocurrent versus V_G for variable E_{PH} (shown as line color). Grey dashed line indicates the color scale cutoff in Fig. 2e.

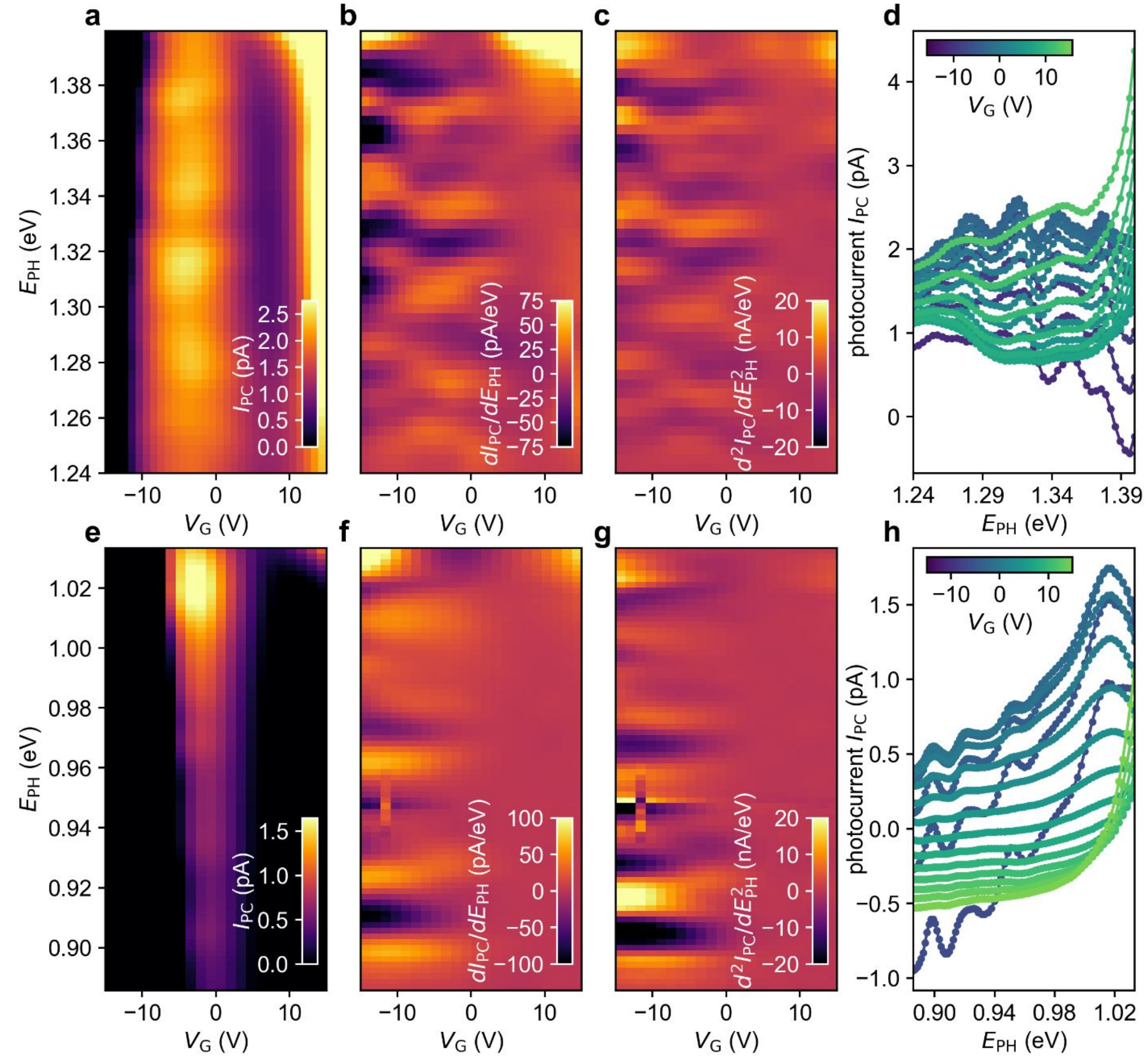

Fig. S4.3 Low temperature photocurrent data. **a**, Photocurrent, I_{PC} versus V_G, near the 1.3 eV direct exciton along with its derivatives, **b**, dI_{PC}/dE_{PH} and, **c**, d^2I_{PC}/dE_{PH}^2. **d**, Line cuts of I_{PC} versus E_{PH} as a function of V_G. **e**, Photocurrent, I_{PC} versus V_G, near the 1 eV indirect exciton along with its first derivatives, **f**, dI_{PC}/dE_{PH} and it second derivative, **g**, d^2I_{PC}/dE_{PH}^2. **h**, Line cuts of I_{PC} versus E_{PH} as a function of V_G.

We now briefly discuss the electrostatic conditions of the device over the gate voltage range studied. In Figure 2, the voltage range shown is within the broad vicinity of charge neutrality, while the charge neutral condition occurs specifically within the narrower region between V_G = -9 V and V_G = 3 V. Fig. S4.3 shows that the sidebands are pronounced across the entire charge neutrality window. There is no marked change of the sidebands as V_G is tuned across the charge neutrality window.

From previous work on vdW p-n heterojunctions (see for example, refs. 23-26), photocurrent in reverse bias - and dark current in forward bias - are expected to be maximal when the device is tuned to the charge neutrality region in the p-n junction. In addition, once the device is doped away from the charge neutral region no more interlayer photocurrent can be measured; at larger voltages away from neutrality the photoresponse is dominated by metallic-like photoconductivity (see our previous transport studies ref. 22 on this material system, as well as Figure 2 in ref. 24). All of the expected behavior for gate-tunable p-n junctions is observed in the work presented here.

In Device 1 (Figure 2), we find that V_G = -3.5 V exhibits the strongest reverse bias photocurrent, and as we tune the gate voltage within the charge neutrality window, we see qualitatively similar sideband behavior. To most clearly exhibit the photocurrent spectroscopy and sideband peaks, we concentrated on line-cuts of V_G = -3.5 V where the photocurrent response is strongest. For other V_G we also find the same periodic photocurrent sideband behavior, see Figure S4.3. From this and additional characterization of Device 2 (Supplement Figure S4.1), we determine that tuning the gate voltage to V_G = -3.5 V results in device response fully consistent with a gate-tunable p-n junction.

This value of the gate voltage where the strongest reverse bias photocurrent is achieved depends on specific details of the device geometry and extrinsic doping, and thus will vary from device to device. As a key distinction, similar behavior is observed for Device 2 shown in Figure 3 (See additional detailed analysis in Section S4.3), yet the charge neutrality window is smaller, occurring between V_G = -0.5 V and 0.5 V. This is consistent with expectation based on the fact that Device 2 has a much thinner dielectric layer (12 nm in Device 2 vs. 290 nm in Device 1), which increases the capacitive coupling of the gate electrode to the device layer. Oscillations as a function of applied source-drain voltage are difficult to resolve as the photocurrent becomes

weaker. This occurs far away for the charge neutrality condition at gate voltages near 1 V and -1 V. This behavior is consistent with the behavior in Device 1, as described above.

S4.3 Data Intensive Exploration of Photocurrent Parameter Space

In this section, we explore the spatial photocurrent response of device 2 in greater detail, showing the MPDPM imaging datasets used to generate the data show in Fig. 3. Fig. S4.4a shows a set of 2,000 photocurrent images versus V_G and V_{SD} with the laser wavelength at 1250 nm (1.0 eV). This is dataset is spatially consistent, i.e. corrected for spatial drift, and from the imaging set we can see the large-scale behavior of the device. Zooming in on a subset of the images in Fig. S4.4b and superimposing the outline of the heterostructure area of the sample, we see that the majority of the photocurrent originates outside the heterostructure, mainly from the semiconductor-metal interface at the device contacts, which will be discussed below. However, near V_G = 0 V we observe distinct and spatially uniform photocurrent originating from the heterostructure area, consistent with our expectation of photocurrent from an interlayer exciton near charge neutrality.

To verify that the photocurrent from the heterostructure is due to the interlayer exciton we take a similar set of spatial photocurrent maps at a fixed value of V_{SD} = -0.35 V while varying the laser wavelength, shown in Fig. S4.5a. Again, we identify the heterostructure region and consider the average photocurrent originating in that spatial area.

The heterostructure responsivity (photocurrent divided by laser power), is shown in Fig. 1. We observed a bright stripe near V_{G} = 0.25 V, corresponding to a resonance in photon energy peaked at 0.96 eV, very close to our expectation of a 1.0 eV interlayer exciton. Importantly, we find that the photocurrent diminishes as the device is tuned away from charge neutrality, indicating that this resonance corresponds to the interlayer exciton, which is consistent with the behavior described in detail in Section S4.2, above.

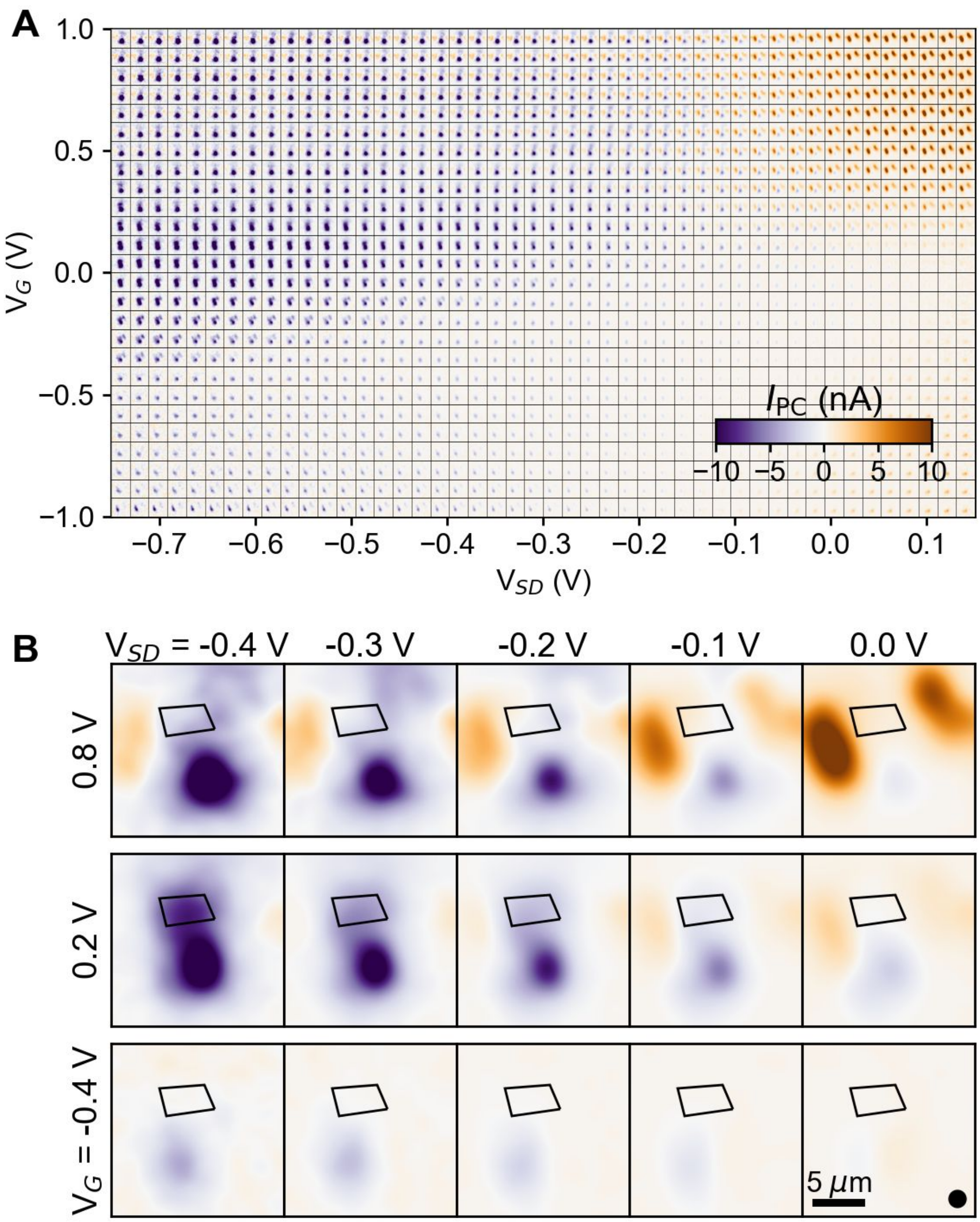

Fig. S4.4 Data intensive imaging of the V_G versus V_{SD} parameter space. **a**, data intensive imaging of device 2 sample at room temperature. **b**, a series of photocurrent images showing the heterostructure area, outlined as a black rectangle.

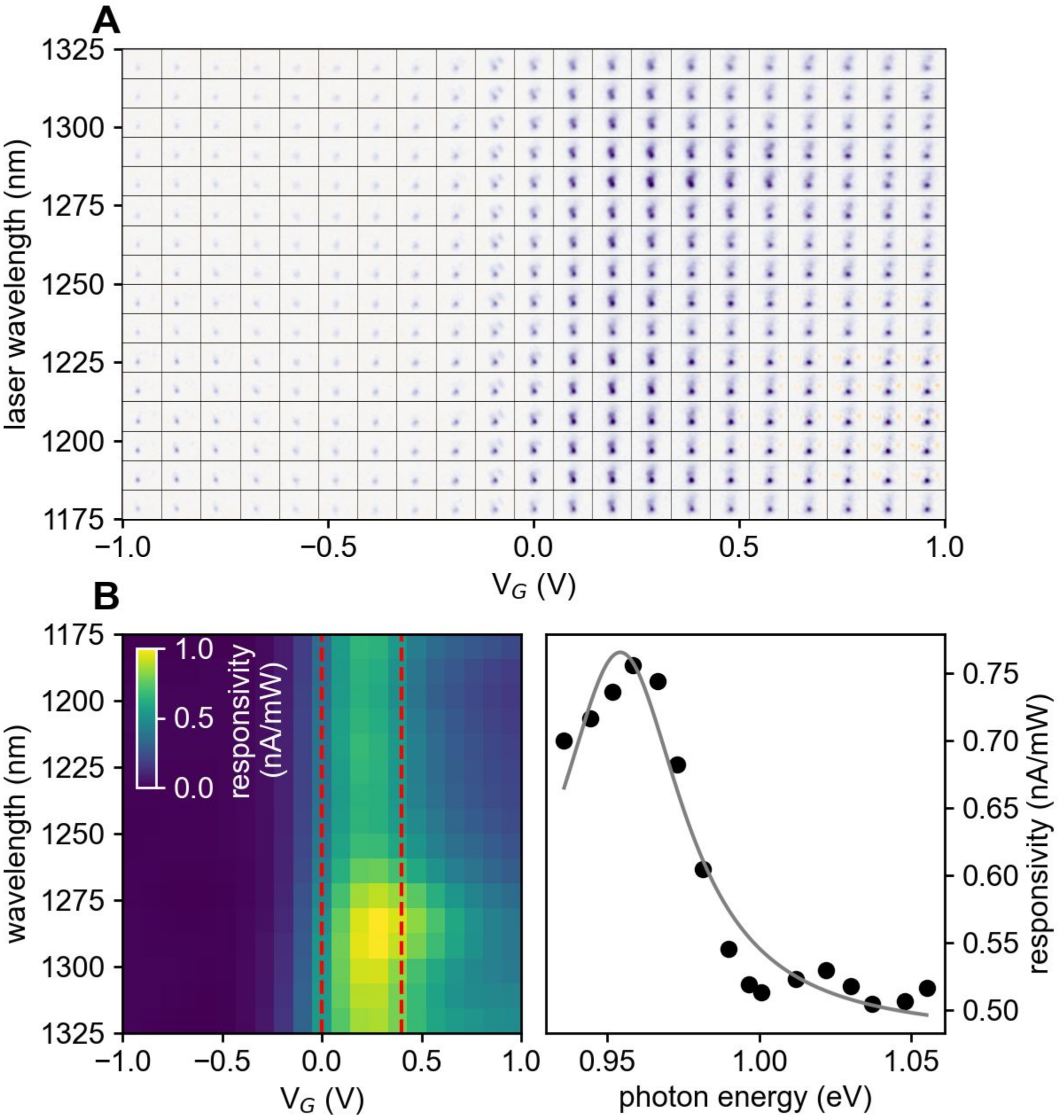

Fig. S4.5 Data intensive imaging of the V_G versus laser wavelength parameter space. **a**, data intensive imaging of device 2 showing photocurrent versus wavelength and gate voltage. **b, left**, Processed responsivity (photocurrent divided by laser power) from the heterostructure regions and, **b, right**, the averaged responsivity (points) fit to a Lorentzian function (grey line).

There are multiple spatially distributed sources of photocurrent in the imaging data sets, the majority of which come from the metal contacts. Fig. S4.6 visualizes the photocurrent at the three points in space corresponding to the principle sources of photocurrent, WSe_2 contact, the heterostructure and the $MoSe_2$ contact. Fig. S4.5a compares a photocurrent map and a reflection image and identifies the locations of the photocurrent as points. The heterostructure photocurrent in Fig. S4.6c has been well described in this work. The WSe_2 and $MoSe_2$ photocurrent in Fig.

S4.6b,d are both more active for positive values of V_G and occur opposite to each other with respect to V_{SD}. Photocurrent at the contacts results from the photo-thermoelectric effect at the metal-semiconductor interface, where the laser produces a local temperature gradient between two materials with significantly different Seebeck coefficients[28].

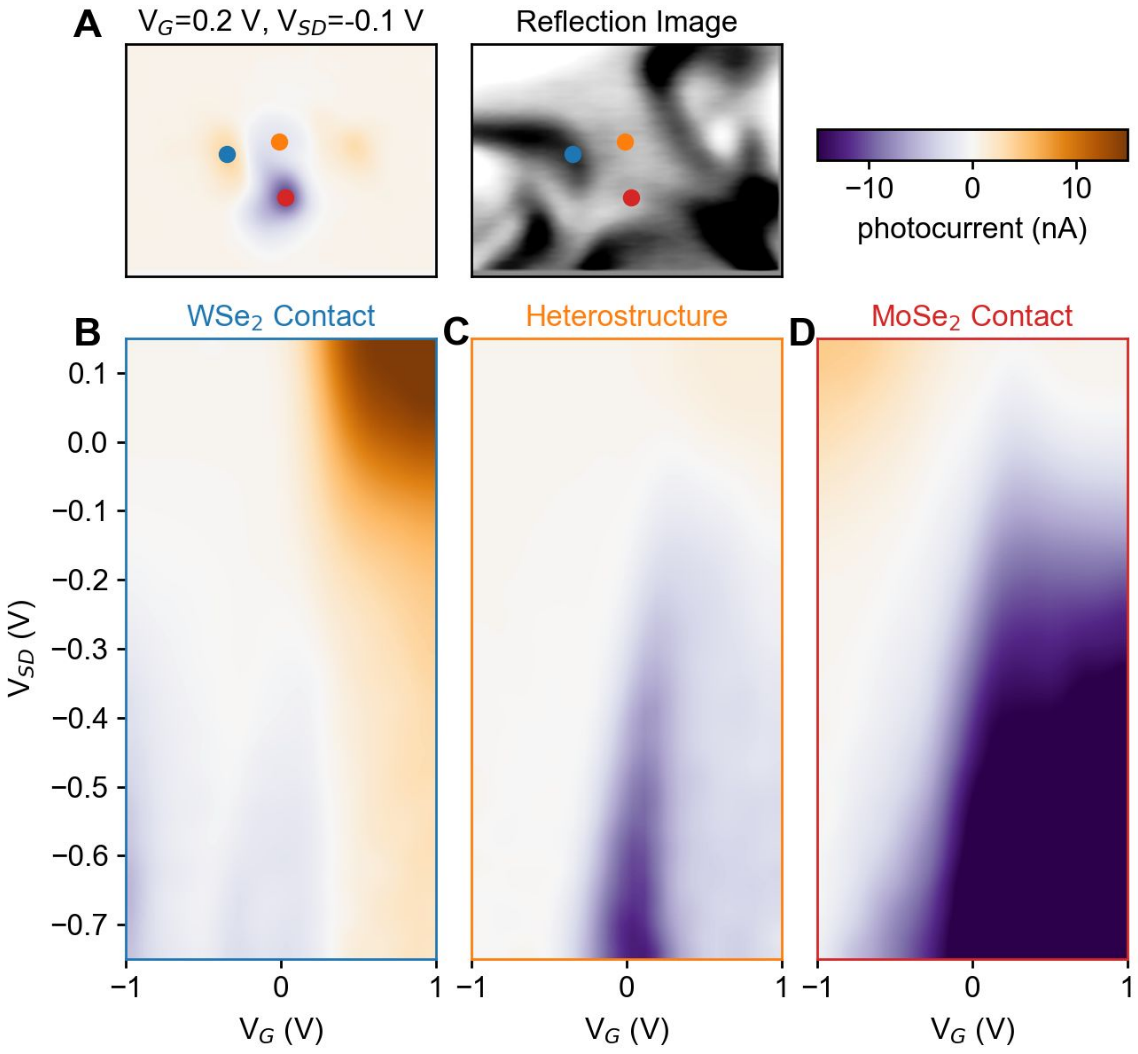

Fig. S4.6 Photocurrent from the metal contacts versus the heterostructure. **a**, Example colormap (colorscale exaggerated) compared to the corresponding reflection image. **b-d**, The photocurrent from the WSe_2 contact, the heterostructure contact and the $MoSe_2$ contact respectively, marked in a with the blue, orange and red points.

S4.4. Analysis of Photocurrent Oscillations in the Current-Voltage Characteristics

In Fig. S4.7a we plot the average heterostructure photocurrent versus V_{SD} and V_{G} and observe a bright feature of positive photocurrent near $V_{\mathrm{G}} = 0$ as a function of increasing V_{SD}. To proceed, we will look at the photoconductance, that is $dI_{\mathrm{PC}}/dV_{\mathrm{SD}}$, which we plot in Fig. S4.7b. We observe a bright feature corresponding to the main feature, but we also see a number of small oscillations in the conductance. To further visualize these oscillations, we take the second derivative $d^2I_{\mathrm{PC}}/dV_{\mathrm{SD}}^2$, shown in Fig. S4.7c, where we see strong oscillations in the photocurrent.

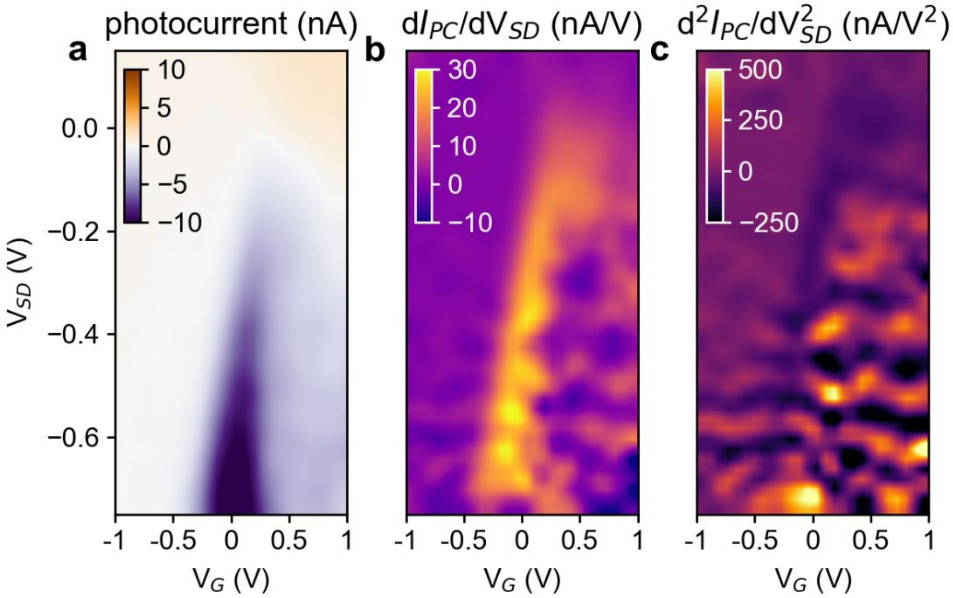

Fig. S4.7 Analysis of the V_{SD} versus V_{G} parameter space. **a**, Heterostructure photocurrent I_{PC} of device 2 versus V_{SD} and V_{G}, calculated by averaging the images in Fig. S4.4 within the heterostructure area. The first derivative $dI_{\mathrm{PC}}/dV_{\mathrm{SD}}$, **b**, and second derivative $d^2I_{\mathrm{PC}}/dV_{\mathrm{SD}}^2$, **c**, with respect to V_{SD}.

In Fig. 3, the approximate energy shift across the heterostructure is obtained by scaling the voltage V_{SD} by two factors: α and η. The phenomenological factor α is obtained from fitting the dark current to the diode equation, $I = I_s(e^{eV_{SD}/\alpha k_b T} - 1)$ (shown in Fig. 1f), while η is a dimensionless parameter that characterizes the dipole length, as described in the main text. By analyzing the heterostructure photocurrent, we find that discretely spaced peaks in the photocurrent spectra emerge near charge neutrality. The interlayer photocurrent extracted from $2\text{x}10^3$

photocurrent images (shown in Fig. S4.4a) occurs predominantly near $V_G = 0$ V, increases as V_{SD} increases in reverse bias ($V_{SD} < 0$ V), and follows the diagonal trajectory (solid line in Fig. S4.8a).

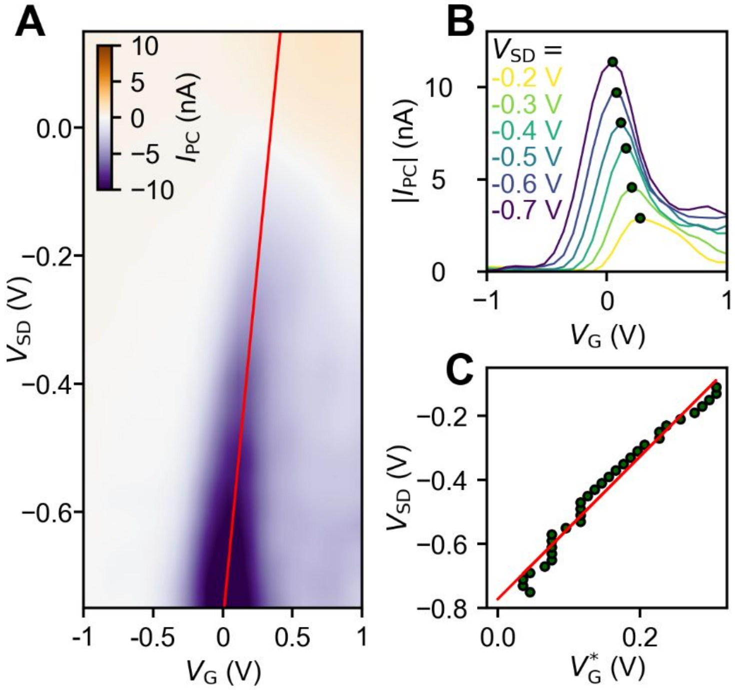

Fig. S4.8 Identifying diagonal self-capacitance behavior in the photocurrent parameter space. **a**, The heterostructure photocurrent parameter space which mainly consists of a bright feature that is diagonal with respect to V_{SD}. **b**, Line cuts of the absolute value of photocurrent versus V_{SD}, green points identify the maxima of the curves. **c**, Shows the V_G value of the photocurrent maximum V_G^* versus V_{SD}. The red line is the linear relation between V_G^* and V_{SD}, which we see tracks the diagonal of the photocurrent in **a**.

By plotting the V_G-dependent characteristics taken at several V_{SD} values (Fig. S4.8b), we extract the gate voltage values of the photocurrent peaks V_G^*, and plot V_G^* as a function of V_{SD} (Fig. S4.8c). Along the diagonal line in Fig. S4.7a, the interlayer electric field is such that the device maintains charge neutrality.

S4.4. Analysis of Photocurrent Oscillations at Different Temperatures

Having now converted the period of room temperature voltage oscillations into units of energy across the heterostructure, we can compare it to the low temperature photocurrent spectroscopy data. Fig. S4.9 compares the low temperature photocurrent 2D-FTs (from Fig. 2) and

the room temperature imaging 2D-FT (from Fig. 3) with the phonon density of states (from Fig. S3.1). We observe that in all cases there is a strong oscillation corresponding to the 30 meV phonon. Remarkably this oscillation is clearly observed even in the room temperature photoconductance oscillations, while less clear in the room temperature spectrum (Fig. S4.5b).

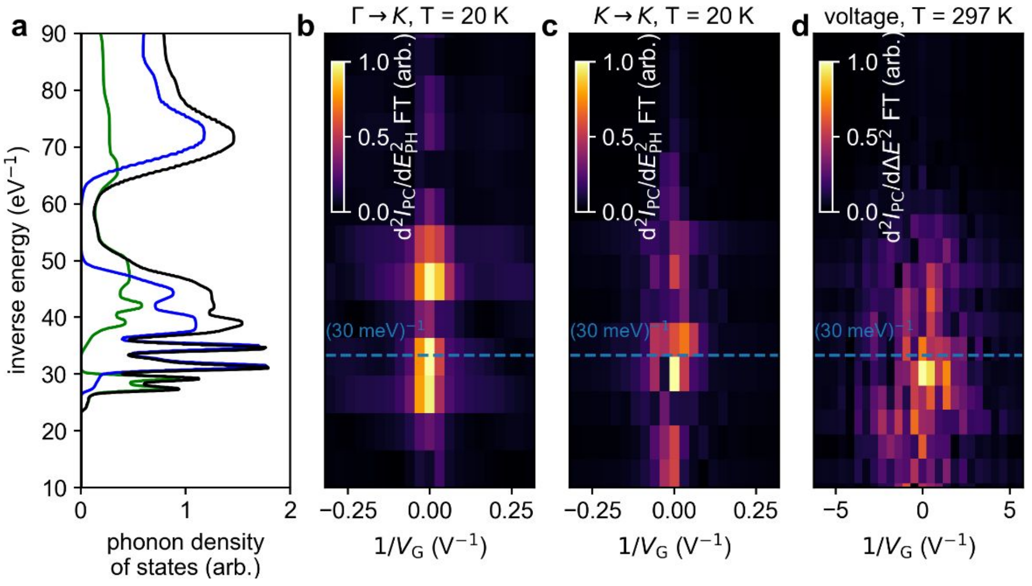

Fig. S4.9 Comparison of photocurrent oscillations across multiple regimes. **a**, the phonon density of states from Fig. S3.1 plotted as a function of inverse energy for comparison with Fourier transforms. **b** and **c**, Fourier transforms of the second derivative of the heterostructure photocurrent of device 1, labeled by the exciton transition they measure. **d**, Fourier transform of the second derivative of the heterostructure photocurrent of device 2. In all cases there is a bright mode at (30 meV)$^{-1}$ marked with a blue dashed line.

The photocurrent oscillations observed in Fig. 3 and discussed above do not occur in the dark current of the system, confirming that they originate from the interlayer exciton. The photocurrent signal is measured using a lock-in amplifier to reject the large dark current. To confirm that the lock-in is functioning properly, and that the oscillations originate from the photocurrent we compare the photocurrent and photocurrent spectrum to the dark current. Fig. S4.10a shows the second derivative of the photocurrent data from Fig. 3 and its Fourier transform;

Fig. S4.10b shows the second derivative of the dark current over a similar range of ΔE and its Fourier transform. Looking at the Fig. S4.10b we note that the dark current is dominated by high frequency noise and is not correlated with the 30 meV oscillation clearly observed in the photocurrent. Thus, we conclude that the oscillation originates from the photocurrent, and therefore the exciton, and not the dark current.

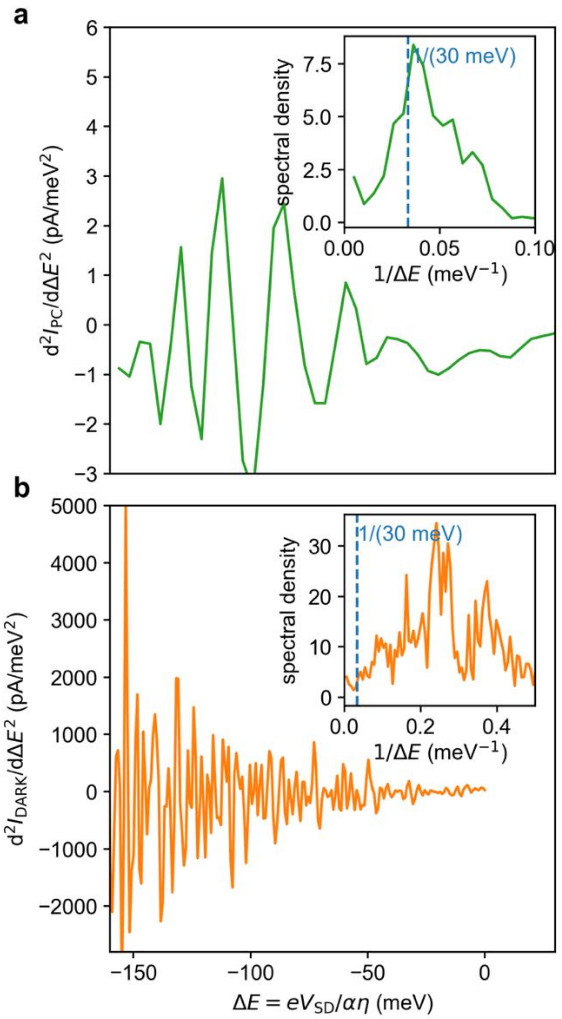

Fig. S4.10 Comparison of photocurrent oscillations to the dark current. **a**, Second derivative of heterostructure photocurrent and its Fourier transform (**a, inset**) from Fig. 3. **b,** Second derivative of dark current, measured with no laser light over a similar voltage range and it's Fourier transform (**b, inset**).

S5 Franck-Condon Transitions and Strong Exciton-Phonon Coupling

In this section, we discuss Franck-Condon transitions, the multiple phonon sidebands, as well as the conditions under which strong exciton-phonon coupling is manifested.

S5.1 Franck-Condon Transitions and Sidebands

We first review how multiple sidebands arise from Franck-Condon transitions and strong exciton-phonon coupling. We begin by analyzing an exciton transition from a ground state, g, to an excited state, e, where the excited state is strongly coupled with the atomic configuration of the system (captured by a phonon coordinate, Q). For simplicity of presentation, we consider a single (optical) phonon mode with frequency ω_0, and its coupling to an exciton mode. The energy surfaces for the ground and excited states can be described by[62]

$$E_g(Q) = \frac{1}{2}A\,Q^2\,, \qquad E_e(Q) = \varepsilon_0 + \frac{1}{2}A(Q - Q_0)^2, \tag{S1}$$

where ε_0 is the bare energy of the transition from ground to excited state in the absence of exciton-phonon coupling, $A = M\omega_0^2$ is an elastic constant and M is an effective mass characterizing the phonon mode. Here Q_0 encodes the relative atomic displacement between the energy minima of the electronic ground and excited state manifolds, which results from the exciton-phonon coupling. For instance, when $Q_0 = 0$, there is no exciton-phonon coupling (Fig. 3a of the main text), yielding an absorption that is characterized by a single broad absorption peak. In contrast, when $Q_0 \neq 0$, exciton-phonon coupling can lead to multiple side-band features in the absorption (Fig. 3b of the main text).

The multiple side-band features can be understood heuristically (see also explicit derivation below) by noting that the atomic configuration, characterized by the phonon coordinates, does not change in the short time over which an electronic transition takes place (the so-called "Franck-Condon principle")[63,64]. This means that optical transitions are "vertical" in the phonon coordinates. Since the energy surfaces are displaced by Q_0, optical transitions originating from the minimum of the electronic ground state energy surface to the electronic excited state energy surface can leave the phonon mode in a range of excited states, thereby producing multiple side-bands. The strength of this effect is captured by the characteristic number of phonons involved in the transition, i.e., the average excitation number in the phonon state after the vertical transition.

This quantity is characterized by the dimensionless Huang-Rhys factor, $S = A\,Q_0^2/(2\hbar\omega_0)$. Interestingly, S also characterizes the depth of the potential well, $\Delta = S\hbar\omega_0$, resulting from the displaced phonon coordinates in the excited state (displaced oscillator).

We now explicitly account for these multiple side-band transitions by considering the vibronic states involved in the optical excitation. The ground and excited state manifolds are characterized by the vibronic states[5,9,62]:

$$\text{Ground: } \left|\psi_g^X, \phi_n\right\rangle, \quad \varepsilon_g(n) = (n + 1/2)\hbar\omega_0, \qquad \text{(S2)}$$

$$\text{Excited: } \left|\psi_e^X, \tilde{\phi}_m\right\rangle, \quad \varepsilon_e(m) = \varepsilon_0 + (m + 1/2)\hbar\omega_0, \qquad \text{(S3)}$$

where $|\phi_n\rangle$ are the modes of the *undisplaced* oscillator (optical phonon mode) in the electronic ground state, while $|\tilde{\phi}_m\rangle$ corresponds to the modes of the *displaced* oscillator in the electronic excited state. Here $n,m = 0, 1, 2,3, \ldots$ are integers, and $|\psi_{g,e}^X\rangle$ are the ground and excited excitonic wavefunctions respectively (For simplicity we assume that the excitonic states $|\psi_{g,e}^X\rangle$ are independent of n,m, as typical for the Franck-Condon principle)[62-64].

Crucially, because the oscillator modes in the excited state $|\tilde{\phi}_m\rangle$ are displaced (in coordinate space, by Q_0) from the those in the undisplaced oscillator of the ground state, their overlap can be non-vanishing even when $m \neq n$. Indeed, the overlap between $|\tilde{\phi}_m\rangle$ and $|\phi_0\rangle$ produces the familiar Poisson distribution[9,62]:

$$|\langle\tilde{\phi}_m|\,\phi_0\rangle|^2 = (S^m/m!)\, exp\,(-S)\,. \qquad \text{(S4)}$$

As a result, the rate of optical transitions in the presence of incident light with energy $\hbar\omega$ follows directly from the golden rule:

$$\Gamma_{g\to e}(m,\hbar\omega) = \frac{2\pi}{\hbar}\left|\langle\psi_e^X, \tilde{\phi}_m\middle|H'\middle|\psi_g^X, \phi_0\rangle\right|^2 \delta(\varepsilon_e(m) - \varepsilon_g(0) - \hbar\omega)$$

$$= \Gamma_0\,(S^m/m!)\, exp\,(-S)\;\; \delta(E_0 + m\hbar\omega_0 - \hbar\omega), \qquad \text{(S5)}$$

where $\Gamma_0 = (2\pi/\hbar)\left|\langle\psi_e^X|H'|\psi_g^X\rangle\right|^2$ is the bare transition rate in the absence of exciton-phonon coupling and H' is the light-matter interaction. This allows for phonons to be involved in the electronic transition between g and e, producing the multiple sidebands as shown in the main text.
